# Supplementary material for: Construction of a High-Density American Cranberry (Vaccinium macrocarpon Ait.) Composite Map Using Genotyping-by-Sequencing for Multi-pedigree Linkage Mapping
Source: G3 (Bethesda). 2017 Mar 1;7(4):1177–89. doi: 10.1534/g3.116.037556 (PMC5386866; doi:10.1534/g3.116.037556)
Supplement: Supplementary file 1 [file 1177FileS1.docx]

File S1. Parameters used in the Tassel v3.0.166 reference-based genotyping-by-sequencing (GBS) pipeline for processing raw sequence data and calling SNPs in the resulting sequence data generated for the parents and progeny of the CNJ02, CNJ04, and GRYG cranberry populations. Identified sequence tags were aligned to the cranberry contigs and scaffolds using bwa version 0.7.8-r455.

| Plugin | Option | Value | Description |
| --- | --- | --- | --- |
| FastqToTagCountPlugin | c | 1 | Minimum number of times a tag must be present to be output. Default: 1 |
| FastqToTagCountPlugin | s | 300000000 | Max good reads per lane. (Optional. Default is 300000000). |
| MergeMultipleTagCountPlugin | c | 3 | Minimum number of times a tag must be present to be output. Default: 1 |
| TagCountToFastqPlugin | c | 1 | Minimum count of reads for a tag to be output (default: 1) |
| SeqToTBTHDF5Plugin | m | internal/04_topm/cranberry_UWisc_Pazaran.topm.bin | TagsOnPhysicalMap (.topm) file containing tags of interest. The -m option i smutually exclusive with the -t option |
| SeqToTBTHDF5Plugin | s | 500000000 | Max good reads per lane. (Optional. Default is 500 |
| TagsToSNPByAlignmentPlugin | y | -y | Use byte-formatted TBT file (*.tbt.byte) |
| TagsToSNPByAlignmentPlugin | errRate | 0.01 | Average sequencing error rate per base (used to decide between heterozygous and homozygous calls) (default: 0.01) |
| TagsToSNPByAlignmentPlugin | mnLCov | 0.1 | Minimum locus coverage i.e. the proportion of taxa with at least one tag at the locus. Default: 0.1 |
| TagsToSNPByAlignmentPlugin | mxSites | 2000000 | The maximum number of SNPs per chromosome for hapmap files (default = 2000000) |
| TagsToSNPByAlignmentPlugin | mnMAC | 999 | Minimum minor allele count. Defaults to 10. SNPs that pass either the specificed minimum minor allele count  (mnMAC) or frequency (mnMAF) will be output. |
| TagsToSNPByAlignmentPlugin | mnMAF | 0.01 | Minimum minor allele frequency. Defaults to 0.01. SNPs that pass either the specifice dminimum minor allele frequency (mnMAF) or count (mnMAC) will be output. |
| MergeDuplicateSNPsPlugin | misMat | 0.05 | Threshold mismatch rate above which the duplicate SNPs won't be merged. Default: 0.05. |
| MergeDuplicateSNPsPlugin | callHets | -callHets | When two genotypes at a replicate SNP disagree for a taxon call it a heterozygote. Defaults to false (=set to missing) |
| MergeIdenticalTaxaPlugin | hetFreq | 0.8 | cutoff frequency between het vs. homozygote calls (default = 0.8) |
| FastqToTBTPlugin | y | -y | output to tagsByTaxaByte (tag counts per taxon from 0 to 127) instead of tagsByTaxaBit (0 or 1) |
| FastqToTBTPlugin | c | 1 | Minimum taxa count within a qseq file for a tag to be output. Default: 1 |
| MergeTagsByTaxaFilesPlugin | s | 300000000 | Maximum number of tags the TBT can hold while merging (default: 200000000) |
| tbt2vcfPlugin | ak | 3 | Maximum number of alleles that are kept for each marker across the population default: 3 |
| tbt2vcfPlugin | mnLCov | 0.0 | Minimum locus coverage (proportion of Taxa with a genotype) (default: 0.0) |
| tbt2vcfPlugin | mnMAF | 0.0 | Minimum minor allele frequency (default: 0.0) |
| MergeDuplicateSNP_vcf_Plugin | ak | 3 | Maximum number of alleles that are kept for each marker across the population default: 3 |
| GBSHapMapFiltersPlugin | mnSCov | 0.8 | Minimum site coverage (default: no filter) |
| GBSHapMapFiltersPlugin | mxMAF | 1 | Maximum minor allele frequency (default: 1.0 = no filter) |
| GBSHapMapFiltersPlugin | mnTCov | 0.1 | Minimum taxa coverage (default: no filter) |
| GBSHapMapFiltersPlugin | mnMAF | 0.01 | Minimum minor allele frequency (default: 0.0 = no filter) |
