# Supplementary material for: Construction of a High-Density American Cranberry (Vaccinium macrocarpon Ait.) Composite Map Using Genotyping-by-Sequencing for Multi-pedigree Linkage Mapping
Source: G3 (Bethesda). 2017 Mar 1;7(4):1177–89. doi: 10.1534/g3.116.037556 (PMC5386866; doi:10.1534/g3.116.037556)
Supplement: Supplementary file 4 [file 1177TableS1.docx]

Table S1. Simple sequence repeat (SSR) markers used to genotype the parents and progeny of the CNJ02, CNJ04, and GRYG full-sib cranberry populations in this study, their primer sequences, their publication of origin, their NCBI ID, and their position (cM) in the linkage groups (LG) of the cranberry composite map constructed herein.

| Locus | LG | Position (cM) | NCBI | Origin | Forward | Reverse |
| --- | --- | --- | --- | --- | --- | --- |
| SCF138607 | 1 | 0.45 | KP278872 | Schlautman et al. (2015) | CATATAGAATACTGGACGGACA | TTCTGCCATCTCCTTTCTC |
| SCF4386 | 1 | 2.9 | KP278612 | Schlautman et al. (2015) | GTTACTCATTTCTTTGCTGAGG | CCTCTTAGTGTTGGAGTTTCAT |
| vm51985 | 1 | 9.43 | JF834280 | Zhu et al. (2012) | TGCTAGTATTTTGACTCAGGTG | GCCTATATATAACCAAGCAAGG |
| vm55441 | 1 | 12.85 | JF834240 | Zhu et al. (2012) | AAAAGGAACACGGATACGAT | GGATTCGAGAACCTATCTCAT |
| SCF3551 | 1 | 18.41 | KP278608 | Schlautman et al. (2015) | CTTCGACGTTTCTGTGACTAT | AGTTGGTGATTGGAAGAGTAAG |
| ct116900 | 1 | 30.57 | KP279112 | Schlautman et al. (2015) | CTCAAACATACCCTTTGAGC | GGTATAGCTTAACAACACACCA |
| SCF118999 | 1 | 30.57 | KP278842 | Schlautman et al. (2015) | CTAAACTCCAAAATGCCTAAAC | AAAGTGGATGGGTTCTAAAAG |
| SCF43220 | 1 | 30.57 | KP278712 | Schlautman et al. (2015) | CTTGTCGAGCATCCTATATTTC | AAAAGTCATGGGAAGGTGTT |
| SCF111370 | 1 | 31.68 | KP278828 | Schlautman et al. (2015) | ACCACATCTTCATTTTGAGC | GTAAAACAATACGGGTCCTTAC |
| SCF124322 | 1 | 31.68 | KP278849 | Schlautman et al. (2015) | TAAAACTGTGAGGTTCAATGTG | CTTCGTGTCTCAAATTACAAAA |
| SCF22962 | 1 | 31.68 | KP278659 | Schlautman et al. (2015) | GTGCAACAGCTAACAGCATA | AGGACCAATACTCAGAACAAAC |
| SCF3914 | 1 | 31.68 | KP278609 | Schlautman et al. (2015) | TGTGGAGTTAGAGTGACATACC | GACAAGAATGATGAGTAGCGT |
| SCF8223 | 1 | 32.27 | KP278622 | Schlautman et al. (2015) | CATTTAGCATCCATCCATTC | GACTGTGGGTTATTCCTTGTAT |
| scaffold_71150 | 1 | 34.07 |  | Herein | GCTTGTGTTCTCTATCCTCTTT | CCTAATACTAACCCCATCCTTT |
| SCF142441 | 1 | 40.43 | KP278879 | Schlautman et al. (2015) | TTGCGTTTACTATCTAAGGAGG | CTCAGCCGTCCAAAAGTAT |
| 162108_K70 | 1 | 40.72 | KP279200 | Schlautman et al. (2015) | GAAGTCGAAACCCTAGCAG | GTCCCTCTCAGTCTCTCACTC |
| scaffold_18824 | 1 | 50.87 |  | Herein | TGTTCTTAGGGAGTTGAGAGAT | TTACCTCAGTCATTCCTCTAGC |
| scaffold_18380 | 1 | 52.06 |  | Herein | CAACCACATAACGCACTACTAT | TGGAGTGATACTTGGTCTCC |
| SCF121995 | 1 | 55.02 | KP278845 | Schlautman et al. (2015) | TAGTCGTGACCAAGAGTGATTA | GCCACCGAGTATATTTCTATGT |
| 242569_K70 | 1 | 55.3 | KP279204 | Schlautman et al. (2015) | GATATGAGAGACGAGGAATCAC | GTCAGTGGACGGTTTTAAGAT |
| 1trimcontig440230 | 1 | 62.75 | KP279249 | Schlautman et al. (2015) | ACACTTTGTAGGTGGTGGTTAT | ATTAGCAGTAGTCCAATCGGT |
| SCF59248 | 1 | 62.99 | KP278740 | Schlautman et al. (2015) | TAGTTGAAAATGGAGAGAGAGC | TTAGATGCCCAACACTACATC |
| SCF102347 | 1 | 64.25 | KP278808 | Schlautman et al. (2015) | GGTAGTGAGCAACGACATAAC | CCTGAAGGTAAAGAAAGTAGCA |
| scf11l | 1 | 76.17 |  | Georgi et al. (2013) | TAATGAGTGCTGGTTCTGCG | TTCAAATCCACGTCAGCAAA |
| scaffold_26291 | 1 | 76.43 |  | Herein | GCCTGTACGATCTTTATCTTCT | TAAAACACTCACCACCCTCTA |
| 1trimcontig238795 | 1 | 77.27 | KP279233 | Schlautman et al. (2015) | AGAGGGAGAGAAGAGTATGGTC | CCGTCAAGATTTGTGAAGAT |
| SCF11186 | 1 | 85.23 | KP278636 | Schlautman et al. (2015) | AGAAAGGCTAAAAGGGTATCTC | GCTCTCAACAACTCGAAAGTA |
| 1trimcontig179737 | 1 | 85.57 | KP279225 | Schlautman et al. (2015) | CCTCCAACTTCTTCATCTTCT | ACTGGTAACTCCTCAGAAACAG |
| vm23232 | 1 | 88.91 | JF834262 | Zhu et al. (2012) | ACAGAGCTCAATGGAGAAAA | TTCTGCTGATAGTGTTGGTACA |
| 1trimcontig239742 | 1 | 92.89 | KP279234 | Schlautman et al. (2015) | AACAAGAACAACTAAGACCACC | TACAAGTTTCAATCAGCCCT |
| SCF149633 | 1 | 92.89 | KP278891 | Schlautman et al. (2015) | CCTTAATACCCATCCCATAATC | CTTCTTTTCATTGTTGTGGC |
| GVC-C347 | 1 | 94.75 |  | Blueberry Markers | GGAGAAGATGACCCAAACGA | AGTCCCTTTGGACCATACCC |
| scf8l | 1 | 94.75 |  | Georgi et al. (2013) | CGAATCCGAAGATCAGAAGC | GGGATACCAGAGATTTCCCG |
| SCF3362 | 1 | 94.93 | KP278605 | Schlautman et al. (2015) | GTACAGCAAAATTCAGCACA | GGATTTATCTACAGCCCATTAC |
| SCF173212 | 1 | 95.35 | KP278924 | Schlautman et al. (2015) | TGTAGTGGGAGATGCTGATAC | AATTGGCGAACTAGAAAGTG |
| SCF30816 | 1 | 96.39 | KP278685 | Schlautman et al. (2015) | GTCCAAAATAGCATCGAAAG | CGCATTACTTCTTCACTATACG |
| 1trimcontig182430 | 1 | 100.35 | KP279226 | Schlautman et al. (2015) | GAAGATGGACCTGAGTAAGAAA | CTACCATTGTGTTCTCAAACTG |
| SCF117157 | 1 | 102.73 | KP278837 | Schlautman et al. (2015) | GGATAGAAACCTGATACGGAC | CGTTACCGTCCCAAATATAA |
| SCF23691 | 1 | 106.91 | KP278662 | Schlautman et al. (2015) | CGGCTTTGTTAGTTGATGTT | CGATGTTGTACTATTCATGTCC |
| SCF39242 | 1 | 106.91 | KP278705 | Schlautman et al. (2015) | ACTCCTGAAGAAGAAGAACAGA | AATGAATGCAGACCACAGAT |
| SCF69981 | 1 | 106.91 | KP278752 | Schlautman et al. (2015) | AGCGTTACCACCGAATATAA | CGAGATATAGTTAAAAGGACGG |
| SCF29735 | 1 | 108.1 | KP278681 | Schlautman et al. (2015) | CGTAAAATCTGTTGTCTCTGTG | TCTCTATGCTCCTTCCACTTAT |
| vm89040 | 1 | 108.1 | JF834248 | Zhu et al. (2012) | TAGACAGACTTTCATGCTATGG | GAACTGATGAAGGTGGTTTATC |
| SCF125768 | 1 | 110.2 | KP279069 | Schlautman et al. (2015) | CTCACTTCTCATACAACATTGG | CACAACAGAACCATCAGTACAT |
| SCF142785 | 1 | 110.2 | KP279083 | Schlautman et al. (2015) | AGGCTCACATTTCTAACTCAAG | ATATCTACCTCCCTAATTTCCG |
| SCF42549 | 1 | 110.2 | KP279018 | Schlautman et al. (2015) | CTCTTCAGCCCTAATCATATTC | CAGGACAAACATCTAGGTCAA |
| SCF208509 | 1 | 111.98 | KP278950 | Schlautman et al. (2015) | GCTTCACACTTGATAGTAGGTTG | TACCGCCATTGTAGCAGAT |
| SCF153722 | 1 | 113.92 | KP278899 | Schlautman et al. (2015) | AGTTATGAGGCTTACGAGGAG | GATGGAACGATGAAACTGAT |
| SCF172019 | 1 | 113.92 | KP278920 | Schlautman et al. (2015) | TGTGAGTAGTTGTTGAAGGGA | CCTCGAAAATCCGGTAAAT |
| SCF68870 | 1 | 115.88 | KP278750 | Schlautman et al. (2015) | GTGAATTGTTGCAGAGTACCTA | TGAGTTGAGTTCATATAGCTGG |
| SCF31394 | 2 | 0 | KP278688 | Schlautman et al. (2015) | GTAGCAAAAGAAGAGACACCAT | CGTTTTCCAGTTCCAGAGTA |
| SCF77145 | 2 | 0 | KP278763 | Schlautman et al. (2015) | TAGAATTAGCCTCCAAGAAGTG | AGAACTAGAAACACGAGAACGA |
| SCF180863 | 2 | 1.11E-16 | KP278930 | Schlautman et al. (2015) | CCAGTTACAGATCCTTGAGTTG | GCAATGTTCCCTCGAATTA |
| 1trimcontig178358 | 2 | 1.79 |  | Herein | AATTGAACGATCCCTATTCC | GATTCATCACCCCTTGAAC |
| SCF22434 | 2 | 1.79 | KP278656 | Schlautman et al. (2015) | TATGTATAGTCCCACAACAAGG | TCCTGTCTATCACTCACATCAC |
| GVC-C722 | 2 | 2.98 |  | Blueberry Markers | AAGTGGATTTCGATTCGGTG | TAATCCCCATCACCGTCATT |
| SCF127382 | 2 | 2.98 | KP279071 | Schlautman et al. (2015) | GTCTTTAGTGCTGGGTTAAAAG | TGATTTCTAGTGTCTCCTCTCA |
| scf28l | 2 | 11.31 |  | Georgi et al. (2013) | AACTCTTCGCTTTGGTTGGA | TCGGTCGTAGAGACGAGGAT |
| SCF74458 | 2 | 11.31 | KP278759 | Schlautman et al. (2015) | GCAGGAAGCTATGATTAAGGTA | TTGAATAGTGTCAGTGGAGAAG |
| SCF965 | 2 | 11.86 | KP278594 | Schlautman et al. (2015) | GTAAACTAACAAGCAACGATCC | GATTTAGCTGATGCAGAGTCAT |
| SCF26049 | 2 | 13.99 | KP278668 | Schlautman et al. (2015) | GTTCAGGTCTGTTGTAAGGAAG | TTTCTTGTAGGACGAAGTGG |
| SCF77376 | 2 | 13.99 | KP278764 | Schlautman et al. (2015) | CTCATCAAAAGAGAGGAGAACT | TGTAACCAATCTTCATGCTG |
| 1trimcontig351427 | 2 | 14.28 | KP279242 | Schlautman et al. (2015) | GACGGCTAAATTGTAACTAACG | AGGGTCCTATCCTATCCTCTAA |
| 314761_K63 | 2 | 14.83 |  | Herein | ATTGTTGGATACTTCATGGC | GTTGGTACTGGTAAACCCTAAT |
| SCF13771 | 2 | 22.53 | KP278642 | Schlautman et al. (2015) | AGGATGATGAAATCTGCAAG | ATCAGTTAGGTGGGGTAAGG |
| 29080_K63 | 2 | 23.92 | KP279160 | Schlautman et al. (2015) | ATGAAAACAGGGTAAACTGG | TCTCAACTCATAGAACTACGGA |
| scaffold_38278 | 2 | 28.18 |  | Herein | TGTATCTTTGATCTGTACGGG | TTCGGGTTAGAGTTTAGTAGGA |
| SCF83079 | 2 | 28.18 | KP279039 | Schlautman et al. (2015) | GTATTCACCAAATCTACCCAGA | GTTAAGGATTGTGTCCCTCA |
| ct130570 | 2 | 29.37 | KP279121 | Schlautman et al. (2015) | GTTCACAATCTGCATCTCCT | ACGTAATAGATCAAGAACAGGG |
| 372875_K63 | 2 | 34.75 | KP279174 | Schlautman et al. (2015) | CACACACAAATCCCAATTTC | GATGGTGTTTTCATAGTTCGAC |
| SCF73288 | 2 | 37.13 | KP278758 | Schlautman et al. (2015) | CAGAGGAACAGCAGACTACAT | CCTAGTACGTCATTGGACATTA |
| CA325 | 2 | 39.13 |  | Georgi et al. (2013) | ACCACCCTCCCATTTCAAAC | AGGCGAAAAAGGTGTTGATG |
| CA325_205 | 2 | 39.13 |  | Blueberry Markers | ACCACCCTCCCATTTCAAAC | AGGCGAAAAAGGTGTTGATG |
| SCF130642 | 2 | 39.13 | KP279074 | Schlautman et al. (2015) | AGGCGGAAGATGAAAGTAAT | TGTCAACATAAAACGATAGCAG |
| SCF158988 | 2 | 39.72 | KP278907 | Schlautman et al. (2015) | CTCTCACCAAAATCACCATTAG | CAAGTATCAAGTTTTAGACGGG |
| SCF48645 | 2 | 40.85 | KP278722 | Schlautman et al. (2015) | AAAATAGGTCCCACATGAGTAG | GCTAGACGATGACACATTATTC |
| ct124256 | 2 | 43.3 | KP279118 | Schlautman et al. (2015) | GCCGTTAGTTCGTGATATGT | CCTACATGCATACGTAAAACAG |
| ct155461 | 2 | 43.42 | KP279141 | Schlautman et al. (2015) | GGTTTCAAACTCGAACAAAG | ATCCTATAACTGGGGATAATGC |
| ct89348 | 2 | 47.79 | KP279100 | Schlautman et al. (2015) | GGCTCAATCTTGTGTAGGTATT | GAGAAAGTGGAAAGATTGTGTG |
| scf2000b | 2 | 50.21 |  | Georgi et al. (2013) | GGCCCTTTTTATCCCCAATA | AATCAAAAGCTGCGAGGAAA |
| SCF158633 | 2 | 52.97 | KP278906 | Schlautman et al. (2015) | AGATGCTGAAGTTTTCCCTT | TATGTGGATTCTTTGCCTTG |
| VCB-C03938 | 2 | 53.56 |  | Blueberry Markers | CCTCAGATAACTGAAACCCGTC | CCTCTCTATTTTCGGTTTCCCT |
| SCF18709 | 2 | 54.15 | KP278651 | Schlautman et al. (2015) | GTAATGGTAAGTGTCGAAATCC | CATAGATGTAACCACGCTTCT |
| 35137_K63 | 2 | 54.47 | KP279254 | Schlautman et al. (2015) | GGAACATCAAAACTCCCATAC | GTTCTTCCCCATTTCAGTAAGT |
| 172672_K70 | 2 | 54.75 | KP279201 | Schlautman et al. (2015) | GATAGTTGTATGCGCTGTAAGA | GTTACCCGAATGAACAGGT |
| 297265_K63 | 2 | 54.75 | KP279167 | Schlautman et al. (2015) | GATCGTCATAACTAAGCTGGAT | GTCTCGAATCACAACAGGATA |
| SCF174468 | 2 | 54.75 | KP278926 | Schlautman et al. (2015) | CAACATTCTTCGCTCACAA | CTAAGAGTTGACATGATTGGC |
| SCF192715 | 2 | 54.75 | KP278942 | Schlautman et al. (2015) | CTCTGCCTTGTTCGTCTCT | AACCAATCGAAGGTGACAA |
| SCF3595 | 2 | 54.75 | KP278607 | Schlautman et al. (2015) | AGACTACAGTGAACAAAGACCA | CTGACTTGGTGTGATTAGTGAG |
| SCF56816 | 2 | 54.75 | KP278735 | Schlautman et al. (2015) | CGGATTGACTAATTTCTGTCTC | CTCTTATTCCACCAAACGAA |
| SCF66692 | 2 | 54.75 | KP278749 | Schlautman et al. (2015) | AAAGTGTATTGGACGGCTG | TTGTTATGGCCCCTCATTA |
| SCF125667 | 2 | 57.45 | KP278852 | Schlautman et al. (2015) | AAGGGAGACATTACACAACAA | TTCGAGATTGACCAAGTATGT |
| scf12916 | 2 | 57.59 |  | Georgi et al. (2013) | GGAGATGGATTTGGCAAGAA | ATCCATGTGGCAGCAGTGTA |
| SCF61078 | 2 | 57.59 | KP278743 | Schlautman et al. (2015) | GACTCTTCATATAACCCACAGC | AAAAGTGCTTGATCGTTAGC |
| CHI03186-1 | 2 | 58.09 |  | Blueberry Markers | TACATCTTGAGGGGCAGTTTTT | GTGGAGTGTGGGATATGGATTT |
| SCF80703 | 2 | 60.43 | KP278770 | Schlautman et al. (2015) | GGTCTTTCTCCTAATCTCCAA | GGAACCCCTAAATAACATACAG |
| 198358_K70 | 2 | 63.49 | KP279203 | Schlautman et al. (2015) | AATCGTCTGTTGCTCAATGT | AACCATACTTACCACAACCAGT |
| SCF213102 | 2 | 63.49 | KP278953 | Schlautman et al. (2015) | GTGAAGATACAGTGGAGAGCA | ATGGTAGTTGTTGACCTGATG |
| 1trimcontig209220 | 2 | 84.19 | KP279228 | Schlautman et al. (2015) | GTATTTGTTCACACTCACCAGA | ACAGTTGTCGAAGCCTCAT |
| SCF142664 | 2 | 85.97 | KP278880 | Schlautman et al. (2015) | TACTGACGATGAGCTAGAGTTG | AATGACAAGTGGAATAGTAGGC |
| contig704 | 2 | 85.98 |  | Georgi et al. (2013) | AAATGGCAGGAATCATGGAC | CTGTTGATCAGCACCACCAC |
| vm54133 | 2 | 87.76 | JF834236 | Zhu et al. (2012) | GTGTAAAATTCCAGGTAGAAGC | ATCAAGCTCTCAGTATCCTCTG |
| SCF122552 | 2 | 88.54 | KP279067 | Schlautman et al. (2015) | TATATCGAGGTCATTGCGA | GAGTTGTCGTTAAGGTTTTGA |
| SCF203038 | 2 | 88.54 | KP279098 | Schlautman et al. (2015) | CACTTCTGTACCCTCTTTTACC | GTCTCATACCTGAATTTTCTGC |
| SCF141794 | 2 | 89.43 | KP278877 | Schlautman et al. (2015) | CCATCTGCATCTATTGTTTTG | CATTTGTAGGTCTATCTTTCGC |
| VCC_B3 | 2 | 90.92 |  | Boches et al.(2005) | CCTTCGATCTTGTTCCTTGC | GTTTGATGCAATTGAGGTGGAGA |
| scaffold_7191 | 2 | 91.81 |  | Herein | ATATAGATGTGTGTGATCGCTG | CTCTCTCCATTTTCCACTAAAC |
| SCF177451 | 2 | 94.19 | KP278929 | Schlautman et al. (2015) | GTACCATATAAGAAAGGGAGCC | CAATAGAAACCCAAGACAACTC |
| SCF175823 | 2 | 97.18 | KP278927 | Schlautman et al. (2015) | AGGGGCAGTTTAGTCCTAGTAT | GCACGTCTTTTCTGTAGTTCAT |
| 1trimcontig332949 | 2 | 99.56 | KP279237 | Schlautman et al. (2015) | ACCCAAACACAAAAGAACAG | GACTGCAAGTGTCTAAATGCT |
| ct118602 | 2 | 99.56 | KP279113 | Schlautman et al. (2015) | TAGAATGCAGTCGTGAAGTGTA | ACTAAATGAGGGGTAGTACGTG |
| Ig15420a | 2 | 99.56 |  | Georgi et al. (2013) | TGGGGGATTTCTCACAAGAG | AATCCCACTTGATTAGGCCC |
| vm13780 | 2 | 100.75 | JF834259 | Zhu et al. (2012) | CCTTCTGCTGGACACTCATA | ACCCATACCAGAGGAGTACATA |
| contig130Fb | 3 | 1.16 |  | Georgi et al. (2013) | GAGATTCTCGCTTTTTCCCC | ATGCACAGCTGCAACAAAAG |
| vm78806 | 3 | 1.16 | JF834245 | Zhu et al. (2012) | CAAAGAAGAGGAGGATTGAGT | GAGCGAGTATTACAAGTGTTTC |
| scf511 | 3 | 4.56 |  | Georgi et al. (2013) | CTCCCTCCTTCCGATGAAGT | CACAAAGTTCCACGCAGAAA |
| SCF90229 | 3 | 5.33 | KP278791 | Schlautman et al. (2015) | GTACTTTTGTGGAACTTAACGC | CTGTCCTTTCACTCCTCTTTT |
| 2ms2a02 | 3 | 6.52 |  | Georgi et al. (2013) | ACCGCAAGAGAGAGATTCCA | GTTTGATGATCACGGTGGTG |
| scf20g | 3 | 6.52 |  | Georgi et al. (2013) | TGAGTGCCGATGAGGTATTG | AGAGGAGGAGACGTGCATTG |
| vm04084 | 3 | 6.52 | JF834250 | Zhu et al. (2012) | GGATTCTCACTCTGATACCATT | GAACGATACACAACGAAGGT |
| vm51409 | 3 | 6.52 | JF834279 | Zhu et al. (2012) | TCCTAGGTTAATTCTTCCCATC | GAGGAGAATCACAAGCTACATT |
| SCF23339 | 3 | 8.22 | KP278661 | Schlautman et al. (2015) | GCAAAACAGAGTTATAGTGGCT | TAGACAGAAGCACAGATTGGTA |
| SCF27755 | 3 | 8.22 | KP278671 | Schlautman et al. (2015) | GAAGTGAGAGTAGGAATCGAAG | CCACAACACAAAACCCTAAT |
| SCF47809 | 3 | 10.77 | KP278720 | Schlautman et al. (2015) | CTTCTACCTTCCAAGATTTGTG | ATTACTATTCCCAGAGACGACC |
| SCF98686 | 3 | 17.2 | KP278803 | Schlautman et al. (2015) | CGTAATTTCACATCCTCGTT | CATAACCAGATAGCACCTCAAT |
| 411475_K63 | 3 | 17.52 | KP279181 | Schlautman et al. (2015) | GCAACAGGGACAGATATTTT | TACGGACTCATAGAAGGTTAGG |
| 42710_K70 | 3 | 17.52 | KP279194 | Schlautman et al. (2015) | GTTACACACACACCCACAGA | GAGAGAGGACTAGGTCGTACAG |
| 1trimcontig440008 | 3 | 17.71 | KP279248 | Schlautman et al. (2015) | GCAACAGGGACAGATATTTT | TACGGACTCATAGAAGGTTAGG |
| SCF57479 | 3 | 19.56 | KP278736 | Schlautman et al. (2015) | AAGTGCAAGTGTGAGAGTGTAT | TGATGGGTGTAAGTGTAAAGAG |
| 121633_K63 | 3 | 23.59 | KP279163 | Schlautman et al. (2015) | GCAGCTCTCTGTAAATTCCTT | ATGGTTGAAGATGTTGATGG |
| SCF38340 | 3 | 25.97 | KP278701 | Schlautman et al. (2015) | CAAACCATTTTAACGGAGAG | AATCATCGTGCATACCTGTT |
| 1trimcontig439466 | 3 | 26.57 | KP279246 | Schlautman et al. (2015) | CGAGTGGATAGTGATGATATTG | ACCAAGAGGAACTACAGGTAAA |
| SCF9815 | 3 | 26.57 | KP278628 | Schlautman et al. (2015) | CATAGGAAGATTGCCTTGAG | GCCTGTTCACATAGATGGAG |
| SCF110888 | 3 | 28.64 | KP278826 | Schlautman et al. (2015) | CTCCTACCCAAATTCACTTGT | CCAAAACTAAACCATTTCTCAC |
| scaffold_37951 | 3 | 30.23 |  | Herein | TTCTGGGTTCCATTACCATA | CTTGTTCTCTATCCTCTTCAGC |
| 82171_K70 | 3 | 33.36 | KP279199 | Schlautman et al. (2015) | TAGTAGAGTTGAAGAGGAGGGA | CTAGGGTTTAAGCAAGCATAGT |
| SCF21119 | 3 | 34.75 | KP278654 | Schlautman et al. (2015) | GGATTTGAGGACTATACCAAGA | TTAAAAGGCATACGCTGAC |
| 1trimcontig436904 | 3 | 35.34 | KP279245 | Schlautman et al. (2015) | TACCAACCACATCACACATC | CTTATGACGATCCCAGTAGC |
| KAN-11440 | 3 | 35.34 |  | Blueberry Markers | CCAGTAACAATGAGCTGCCA | GATCGTTGCTGAAGGGTTGT |
| SCF108252 | 3 | 35.34 | KP278817 | Schlautman et al. (2015) | CCTATGTAATTGGATTCTACCC | GTGTATCAAGGTGGAGAAAGTC |
| SCF128992 | 3 | 35.34 | KP278858 | Schlautman et al. (2015) | GAGTGTTGAGTTATAGGGGTTT | TCACAAGAATAGAAGGATGGA |
| SCF22477 | 3 | 35.34 | KP278658 | Schlautman et al. (2015) | CTCTCCCCTACTTTCTTCCTAT | GCCGCTAACACAATTAACTAAC |
| SCF53750 | 3 | 35.34 | KP278727 | Schlautman et al. (2015) | GTTTCATAGAGATGGGTTTCTG | CTTGGTTCCCTAAGCTACATT |
| SCF78184 | 3 | 35.34 | KP278766 | Schlautman et al. (2015) | CACATTTAAGAGCTACCACCTT | GGTGAAAGAGAAGACTGGATT |
| scf9025 | 3 | 36.53 |  | Georgi et al. (2013) | TGGCTCCTATAGCGTGTCCT | GCACACCAGGTTCCTTGATT |
| SCF7845 | 3 | 38.92 | KP278620 | Schlautman et al. (2015) | GTTCTGACTATTGTGATGGGTT | TGCAATGAATACTGGAAGTG |
| SCF149145 | 3 | 54.52 | KP279087 | Schlautman et al. (2015) | CTTCAACATATACCCACCCTAT | GACCAAAACTAGAAAACTCCCT |
| scf5304 | 3 | 56.18 |  | Georgi et al. (2013) | TACACAGCTTCATTCGGCAA | AAGCTCACCCAATCGAAAGA |
| SCF85773 | 3 | 58.58 | KP278780 | Schlautman et al. (2015) | TCTTGAACACAGCACAACAT | ATAAGTTTGCCCCTTTTGTC |
| scaffold_13275 | 3 | 60.96 |  | Herein | AGGAAGAGTGATATTAGCGGT | GAGTATGTGTTTTGGTTGTGG |
| scaffold_31246 | 3 | 60.96 |  | Herein | GGGATATTACACACACACACAC | AGGAGAGAGAGTACCAGTCTTTT |
| scf10688 | 3 | 60.96 |  | Georgi et al. (2013) | TCACTTTTCTTTCATGCCCC | GTGCTCCCACTAGCCCATAA |
| scf142e | 3 | 60.96 |  | Georgi et al. (2013) | CTACCGAGCTGGTTGAGGAG | CGAGCGCATAATCATCTTCA |
| ct155339 | 3 | 62.75 | KP279140 | Schlautman et al. (2015) | AAGTTCCTCTGTTACAAGCTCT | ATGACGAACTCTTCCTCCTTAT |
| SCF177450 | 3 | 62.75 | KP278928 | Schlautman et al. (2015) | TCTAAAACTCTCCTCTCACCTC | GATAGCAGTGGACTCATGTCT |
| 313711_K70 | 3 | 68.63 | KP279215 | Schlautman et al. (2015) | CGACTTAATCCCTCTCTTTCTA | CTTTACTTTTCCATCTCCCTC |
| ct89711 | 3 | 70.52 | KP279102 | Schlautman et al. (2015) | CTCCACACCCACAATCTG | CGTCTTATTTTTAGTCACCTGG |
| SCF157992 | 3 | 70.52 | KP278904 | Schlautman et al. (2015) | TAGGTTTGTCTCTTATCCATCC | GAGTTTGTGATTTCTTAGGAGC |
| scaffold_19017 | 3 | 74.7 |  | Herein | GACTACTCTTGTCTTCGATTGG | GTATCAAAATCTCTCTTGGGC |
| SCF153094 | 3 | 74.7 | KP278897 | Schlautman et al. (2015) | TGTCATTAGGGTTCCTCAAA | CACCTAGACAACATCGAAACTA |
| contig480Fb | 3 | 82.61 |  | Georgi et al. (2013) | GATGATGTGGGGCCTAAGAA | CGCATTCGACTCAATGTTGT |
| 1trimcontig443603 | 3 | 83.66 | KP279251 | Schlautman et al. (2015) | TGCACCTCCTCTCTCTCTAA | GGTTATGATGGTGGGAAAG |
| SCF162565 | 3 | 85.17 | KP278913 | Schlautman et al. (2015) | CTTCCGTGATTGTTCTTGTAG | ACACAGATGGGATGTTGTATC |
| NA172 | 3 | 87.55 |  | Georgi et al. (2013) | CCTCGTCCTCCTCTTCCTCT | GTTTGACTTTGGAGAAGGCGAAG |
| scaffold_35862 | 3 | 87.55 |  | Herein | GGAAGATAGAAAACACGACAAG | GATGATTACCCTCCTCTCTCAT |
| SCF58861 | 3 | 88.74 | KP278738 | Schlautman et al. (2015) | GTTGACTAAAAGGCATTGGA | GACTACTATTTTCTGCACAGGG |
| SCF27510 | 3 | 92.4 | KP278670 | Schlautman et al. (2015) | CCTTCAGATTCAACGTATTCTC | GGTGTATCACATCCCAAAAC |
| 418294_K63 | 4 | 0 | KP279189 | Schlautman et al. (2015) | CAAGAACAAGAAGAAGAAGACC | AGAGACCACCCAAAAGATAAG |
| SCF56561 | 4 | 0 | KP278732 | Schlautman et al. (2015) | ATTAGCCATTCGTGATTAGG | TAAGGAGATACGACCAAGAAAC |
| 1trimcontig176303 | 4 | 0.59 | KP279222 | Schlautman et al. (2015) | GCTGATTAGGTTCACTTTCTTC | TTTCTTCACCTCTTTCTCTCTC |
| SCF26697 | 4 | 0.59 | KP278669 | Schlautman et al. (2015) | TCGTAACTATTCAGTGGGTGT | GGAGCAGTAGAGATTAAACGAC |
| 2ms4d10b | 4 | 1.19 |  | Georgi et al. (2013) | GGAAACGATGCCGTTTTCTA | CAACCCTTCCAGGTCAAAAA |
| ct119523 | 4 | 1.19 | KP279114 | Schlautman et al. (2015) | GACTCATGGGAGTGAGGAC | TGAACTTGTGTAGTCTTTACCG |
| ct94504 | 4 | 1.19 | KP279105 | Schlautman et al. (2015) | CTCTAAAGCTCAAGAAAACGTC | AGCTGTGACTATAAGGGATTTG |
| GVC-V24d10b | 4 | 1.19 |  | Blueberry Markers | GGAAACGATGCCGTTTTCTA | CAACCCTTCCAGGTCAAAAA |
| SCF83036 | 4 | 1.34 | KP278776 | Schlautman et al. (2015) | CAACAGTCCTCAAAATCACTC | GTGAACAGAAGTAGAGATCGG |
| scf105g | 4 | 2.39 |  | Georgi et al. (2013) | TCTGTACCTCCCCATTCCTG | CCAAACACGCCGTTAATCTT |
| vm83024 | 4 | 4.18 | JF834247 | Zhu et al. (2012) | TTCGCCTCTCTAGTTTCTAGTC | GTTATATTACCACAAGCACACG |
| SCF162175 | 4 | 7.16 | KP278912 | Schlautman et al. (2015) | ACACGTTGAGGTTCCAAAT | AGTTTCTGATTGACCTAGATGG |
| SCF9157 | 4 | 7.16 | KP278626 | Schlautman et al. (2015) | GGCTTAACAAATTAGCCCTT | GAGAGGATTTACCGACAAAGTA |
| scf1172 | 4 | 10.82 |  | Georgi et al. (2013) | GGGGTTTGTGTGTTTATCGC | GTATGCGAATTCAAAGCCGT |
| 416328_K63 | 4 | 15.6 | KP279185 | Schlautman et al. (2015) | GTATGCCCAGAATATCCATTAC | TAGTCACGAGGAAAGCTAAAGT |
| ct119590 | 4 | 15.6 | KP279115 | Schlautman et al. (2015) | ACATGACATCAATTGCCC | TATCCTACCTCAAAGAGCCTAA |
| SCF113389 | 4 | 17.98 | KP278831 | Schlautman et al. (2015) | GACATCACTCAAGCAAGATAAA | CCTCGATTCCTCAAGATATG |
| SCF72229 | 4 | 17.98 | KP278756 | Schlautman et al. (2015) | CAACTTCTACAACCACTCCAC | GATTTATTGTGCTACACTGGTC |
| 5ms2b12 | 4 | 18.45 |  | Georgi et al. (2013) | AAAACTGCAACTGGAATCGG | GTCTGCAGGTCACAGGTTCA |
| CA855F | 4 | 20.23 |  | Boches et al.(2005) | CGCGTGAAAAACGACCTAAT | GTTTACTCGATCCCTCCACCTG |
| SCF140628 | 4 | 20.23 | KP278876 | Schlautman et al. (2015) | GTGAAATTGGTCAGGTTGAT | GTCGTCATCATCATCTCCTC |
| SCF46739 | 4 | 20.36 | KP278716 | Schlautman et al. (2015) | ATGTTAGGTGATGCTGTTGTC | CAGGTGCTTATTTTCGTTTC |
| vm52682 | 4 | 23.94 | JF834282 | Zhu et al. (2012) | CTCAGGTTATCAGGCTTATTTC | CAATTAGTGTGTTCCCAACTC |
| SCF6530 | 4 | 26.79 | KP278614 | Schlautman et al. (2015) | CCCCAAGTATAATGTGTAAAGG | AGTTCGCATAGAAACTGTAGGA |
| SCF124075 | 4 | 29.33 | KP278848 | Schlautman et al. (2015) | ATTTTCCCTCCAACCTCTAT | GGTGCAACCAACTAACATAA |
| scf6955c | 4 | 29.89 |  | Georgi et al. (2013) | ATGCCTGCCATTCATCATTT | TTTCCGTCATTTCGTCCTTC |
| vm52204 | 4 | 29.89 | JF834281 | Zhu et al. (2012) | CTATATAACAGACGTCCAACCC | GGGTTGTTTCAGACAAGTAAGT |
| 305731_K63 | 4 | 32.32 | KP279169 | Schlautman et al. (2015) | GATTTCTTCGTGTTTCTCTCTC | TGCCTTTCTCTACTCTCTCTTC |
| scaffold_24341 | 4 | 32.32 |  | Herein | CTGTTTCAGGTTAGCATTGAG | GAGTTTTGCAGTAGCTCTAGGT |
| SCF28100 | 4 | 32.32 | KP278674 | Schlautman et al. (2015) | TAGAAACTAACATGGGAGGTGT | GCACGCTGTATTGATAGAAGAT |
| scaffold_120214 | 4 | 32.91 |  | Herein | GTGGACCTCGACTATCATTATT | TTCAAATACTCCTGCTGACTAC |
| SCF154541 | 4 | 35.64 | KP278900 | Schlautman et al. (2015) | AGAAAGCACAGTAGGTATGGAG | CAAGAAACCCTAGAGACCAAT |
| SCF38553 | 4 | 39.4 | KP278703 | Schlautman et al. (2015) | CTTCTGTTTACTCACTTCCACC | ATGGTCCCAAGATACTTTAGC |
| SCF15845 | 4 | 41.47 | KP278645 | Schlautman et al. (2015) | AGGCTAATGAAGAAGAAGTCTG | GACCAAGACAAGATGAACAAG |
| SCF194552 | 4 | 45.88 | KP278944 | Schlautman et al. (2015) | CACAGGTGTAGGGTCTTGTT | AAAAGGAGGCAAGGATAGAG |
| SCF16359 | 4 | 56.42 | KP278647 | Schlautman et al. (2015) | GAAGTGCTTTTCTTTCGTAGAG | AGACAGATTAAGATCCACCTTG |
| SCF96539 | 4 | 56.9 | KP278800 | Schlautman et al. (2015) | GTAGCATAACCACCTCTTATCC | ATCTTGATGACTGTGTAAGCTG |
| NA1713 | 4 | 59.88 |  | Georgi et al. (2013) | ATTCGCGTATGGAAGGTGAC | CTCACACCACTGTGGCTCAT |
| SCF100820 | 4 | 59.88 | KP278804 | Schlautman et al. (2015) | GTAATTCCACTTAACCCACTCA | GTTGAAGATAAACCACCTTCC |
| scaffold_3314 | 4 | 62.26 |  | Herein | CAGATTAAGGAAAAGGAGAAGG | AAAGACCAACCTAGTCCACATA |
| scaffold_51535 | 4 | 62.26 |  | Herein | GACCAATCAGAATTAGACAACG | GTTCGTGAAAATAGGAGTACGA |
| scaffold_5180 | 4 | 62.26 |  | Herein | CTCTCTTACTTTCCACTGTTCC | CTCTATCCTCTTCAACACCACT |
| SCF204979 | 4 | 62.26 | KP278949 | Schlautman et al. (2015) | GGAAAGAGGTAAGAAATGGG | TAAGAGTTCCCACAACCAAA |
| scf24k | 4 | 62.26 | JN230516 | Georgi et al. (2013) | ATTGAGCCCCACACTACAGG | AGCCATGGAAATCCAACAAA |
| SCF79014 | 4 | 62.26 | KP278767 | Schlautman et al. (2015) | TCTCTGTCTCTGTCTCTGTCTG | CCAAATCAAGGTCTGTCTATCT |
| SCF101064 | 4 | 62.31 | KP278805 | Schlautman et al. (2015) | CATCAGACAGAAAGCAGTTAAG | CCCCAAGTATATTAGCAAACAC |
| contig600 | 4 | 69.82 |  | Georgi et al. (2013) | GCCAAAGCTGGAGAGAGAAA | GACTTCAGCAGCCAACATCA |
| SCF49598 | 4 | 70.59 | KP278723 | Schlautman et al. (2015) | ATGAGGTTTTCCAACACAAC | TCAGAGGGAAGTACATGAGAAT |
| SCF108294 | 4 | 77.19 | KP278818 | Schlautman et al. (2015) | GGTAAGATTGAGGTTCTGGTCT | GGTAGAAGCAAGAAGATGCAC |
| SCF11084 | 4 | 77.19 | KP278635 | Schlautman et al. (2015) | GTTGGCTGAGGTAGCTGATAG | CCTAAAAGGGCTCACAAGTTA |
| SCF1128 | 4 | 77.19 | KP278597 | Schlautman et al. (2015) | GTTTGTTGTTGTGGTGGTTT | CCTTACTTGACGCTTACTTCAG |
| SCF46912 | 4 | 77.19 | KP278719 | Schlautman et al. (2015) | GAACAATAAAGAGGCTAGAGGA | CATAGTTGTAGAGAAGATCGGG |
| SCF53282 | 4 | 83.32 | KP278726 | Schlautman et al. (2015) | GACAATCACATACCCATAACAG | CCACTCTTTCCCTCTATCG |
| SCF18363 | 4 | 84.57 | KP278650 | Schlautman et al. (2015) | CAAAGACCGCTAGGTTTACA | ACTGCTCACTAGACAAGATCG |
| SCF145739 | 4 | 85.1 | KP278886 | Schlautman et al. (2015) | AAATCCTCCTGTTTTAGACTCC | CCTCAAGTCATCATTCCCT |
| 281884_K70 | 4 | 85.7 | KP279207 | Schlautman et al. (2015) | TCCACTATCTTTAGAATCCCAC | AGAGGATGGAGTTCCTTGATA |
| scf26r | 4 | 85.7 |  | Georgi et al. (2013) | ATGATGTTGGATGTGCCTCA | TTCCTCAACAAACCCTCCAC |
| SCF29560 | 4 | 85.7 | KP278680 | Schlautman et al. (2015) | GTGTGGTGTGGTCTCTACAAT | ACATCTCTTTGGCTGATACTTC |
| SCF74895 | 4 | 85.7 | KP278760 | Schlautman et al. (2015) | GTACTCCTCTCCGTCTAGCAT | GATTTTATGCGTTAGCTCCA |
| SCF14119 | 4 | 86.35 | KP278643 | Schlautman et al. (2015) | TAACAGTACAATGCCTAGTTCG | GGATTCTCTTGCTTTGGTATAG |
| SCF51810 | 4 | 86.35 | KP278725 | Schlautman et al. (2015) | TATTACTCTGTTGCTGCTGTTG | ACTAAACCCTAATGTCCCTTCT |
| 251788_K63 | 5 | 0 | KP279166 | Schlautman et al. (2015) | GATCTTTACCACTCCCCACT | GGATTCTCTGTCCATTGTTG |
| ct153008 | 5 | 0 | KP279136 | Schlautman et al. (2015) | CTTTCCAAGATCTTCATAGGC | CGACAGTATAATAGCATGGAGA |
| SCF143318 | 5 | 0 | KP278883 | Schlautman et al. (2015) | CCGTGCTTAAATTCTGTAGTG | TCATCCATAGGAGAACATCC |
| SCF28613 | 5 | 0 | KP278677 | Schlautman et al. (2015) | CATTCTTCACTCCAACTTCAG | CAAGTCCCATCATCATTTTC |
| SCF804 | 5 | 0 | KP278592 | Schlautman et al. (2015) | CAGTCAACAGAGAATACACCAC | TTCCCTATGAAAATCCACAC |
| 308839_K70 | 5 | 0.29 | KP279211 | Schlautman et al. (2015) | ATAATGTGTCCAGTCCCTTTC | TTCCTTCCTCAATCCACTC |
| SCF7132 | 5 | 8.58 | KP278616 | Schlautman et al. (2015) | AAGGGGAAGGACAATAAGAA | AATTTGATGACTGTTGTGGC |
| ct144370 | 5 | 9.77 | KP279127 | Schlautman et al. (2015) | GTAGGAAAAGTTTGAACCGTC | TCAAAGGTTTCACGTTTCTC |
| SCF32727 | 5 | 12.51 | KP278689 | Schlautman et al. (2015) | ATGTAACGGTCTCCACTTTCT | TAGTATCTTCGTGGTCAGAGGT |
| SCF59035 | 5 | 15.21 | KP278739 | Schlautman et al. (2015) | AGATTTTGAACGATGTCTGC | GATCTATCGCTTATCCAGTACG |
| SCF259 | 5 | 23.41 | KP278591 | Schlautman et al. (2015) | TGACAGTACCAATAGCAGGAC | AACACCCAGTCGTTATACATCT |
| SCF31208 | 5 | 25.19 | KP278687 | Schlautman et al. (2015) | AACAGCACCACTACAACACTT | AGAGAACAATCGTCTAATCGTC |
| SCF132595 | 5 | 25.48 | KP278863 | Schlautman et al. (2015) | CAAACAAATCTCAACAACACC | ATTTCAAGATAAGCTCTCCACC |
| SCF97378 | 5 | 31.09 | KP278801 | Schlautman et al. (2015) | GTAGAGATCGTTGTCGTCATTT | AACATCGTGGTGTATTGGAT |
| 3ms2g09 | 5 | 37.05 |  | Georgi et al. (2013) | CCTAAATTGCAGCCACTGGT | ACGGCAAGACAACGTTCATT |
| vm39030 | 5 | 37.05 | JF834273 | Zhu et al. (2012) | CTGATTACTGAGTCTACTAACACCA | ACAGATTTGTAGTCACGAAGTG |
| ct98042 | 5 | 47.12 | KP279108 | Schlautman et al. (2015) | CCTTTTAAGTACTTTCCCTTCC | CCCCTCATCTTTATGTGC |
| scaffold_29411 | 5 | 47.12 |  | Herein | TCTTACTGAATGCTCTTAGGGT | CTCATTACATCAGCTTGTTAGC |
| SCF149976 | 5 | 47.12 | KP278893 | Schlautman et al. (2015) | TATACCCATGTATGTACGCATC | ACTCTAAGCAGGACAATGCTAT |
| SCF8987 | 5 | 47.12 | KP278625 | Schlautman et al. (2015) | AATCTTTGTCTGAGGTAAGTGG | AACCAGTGTAGTGCAGTTTATG |
| SCF83615 | 5 | 48.65 | KP278777 | Schlautman et al. (2015) | ATTAGTCGATCTCCTTTTCCTC | AAATTGTAGAGCCAACACTAGG |
| SCF46751 | 5 | 51.89 | KP278715 | Schlautman et al. (2015) | ACCAGATGAAGAAGAAGAAGC | GCCTCTCATTACCATTACAAAC |
| SCF101878 | 5 | 54.27 | KP278807 | Schlautman et al. (2015) | GACTCATTGGATACGTGCT | TCTATGTAGCTTTGAAGTGAGG |
| scf9e | 5 | 58.41 |  | Georgi et al. (2013) | TCACAGCGGAGAAGTTGATG | ATTTGCGAATCAACCCAAAC |
| SCF9068 | 5 | 61.82 | KP278624 | Schlautman et al. (2015) | AAATCTAGGTAGGAGCAGGTCT | ATGGAGGAGGAGATATGTGAT |
| 1trimcontig237406 | 5 | 63.17 | KP279230 | Schlautman et al. (2015) | TCTTAGGAAAGACGAGAACATC | GAAAGGAAGGTATGCTACAGTT |
| 308812_K70 | 5 | 63.17 | KP279210 | Schlautman et al. (2015) | GAAAGGAAGGTATGCTACAGTT | TCTTAGGAAAGACGAGAACATC |
| SCF11431 | 5 | 63.17 | KP278637 | Schlautman et al. (2015) | GCTGCTGATTTGTTATGTAGAG | CACTTAGCCCCTTAAACTATTG |
| SCF88902 | 5 | 63.17 | KP278785 | Schlautman et al. (2015) | GTGTTGTAGGATGAACCGAT | GATTTCCAGCATTTGATCTC |
| SCF6195 | 5 | 68.53 | KP278613 | Schlautman et al. (2015) | GACTATGAATCTGACGCTCAC | CCAGTAAATACGTGACTAATCG |
| 1trimcontig191066 | 5 | 73.72 | KP279227 | Schlautman et al. (2015) | GATATTAGTCCGGTTTACGAGA | GATACAGGAGTCGAGAATGAAT |
| vm07778 | 5 | 75.97 | JF834253 | Zhu et al. (2012) | ATATACGTACACTCACGCACAC | GTTAGGTGCATAATAACGGTTG |
| ct115258 | 5 | 79.37 | KP279111 | Schlautman et al. (2015) | GTTCGTTGTGGAAGTCACAT | CAAAATGAGTGCCAGATAGTG |
| 47166_K70 | 5 | 88.31 | KP279195 | Schlautman et al. (2015) | TATTGAGAGTGTGAGACCGTT | TGGTAAGTATCGTAGGTCCAAT |
| CA794F | 5 | 88.31 |  | Boches et al.(2005) | CGGTTGTCCCACTTCATCTT | GTTTGAATTTGGCTTCGGATTC |
| SCF108454 | 6 | 0.9 | KP278819 | Schlautman et al. (2015) | CTAACTAAATGAAGTGTTCCCCT | ATGTCACGCTCTGAAGTTTG |
| SCF192219 | 6 | 0.9 | KP278941 | Schlautman et al. (2015) | GAATTTTGTCGTTCCAGAGA | AAAAGAAGAAGAGGAATGGC |
| SCF79620 | 6 | 0.9 | KP278768 | Schlautman et al. (2015) | TAATAGCCCTTATACCTGCACT | GAGCATAGACAGCATACAAAAG |
| 1trimcontig339726 | 6 | 2.19 | KP279240 | Schlautman et al. (2015) | TACTCATGTCGAAGCAATAGAG | CTTTAGCAGAGGAGAAACAAGT |
| SCF109269 | 6 | 2.19 | KP278820 | Schlautman et al. (2015) | CACTCCTTCCTTATAGATCAGC | AAGTAGAAGAGCAGCACAAGAG |
| vm31701 | 6 | 2.78 | JF834268 | Zhu et al. (2012) | GTCACTGGTAATGCTATTCTGA | CTTCTTTGTTTCATCTCCCTAC |
| SCF124927 | 6 | 3.02 | KP278850 | Schlautman et al. (2015) | CGAGTGTCATTAGCAACAGA | TATCACTTTAGATCGAGCAGAC |
| GVC-V22a02 | 6 | 3.38 |  | Blueberry Markers | ACCGCAAGAGAGAGATTCCA | GTTTGATGATCACGGTGGTG |
| ct92708 | 6 | 11.05 | KP279103 | Schlautman et al. (2015) | CCCTAGATATTTCTGGAACACT | AAGATAGAGAGAGACAAAGGAGG |
| GVC-V31e03 | 6 | 11.05 |  | Blueberry Markers | GGCACCGACGTACCCAC | GGGTGAGTAAAGGACGGTGA |
| SCF113558 | 6 | 11.05 | KP278832 | Schlautman et al. (2015) | GAGCTTGATCTGGGTATCTTT | CAAAATCAGAATCGACTGC |
| SCF147117 | 6 | 16.85 | KP278888 | Schlautman et al. (2015) | AGATATGGAGTGGATTAGGTTG | GTTAGAGTGAAATGAGCCCTAT |
| scf283b | 6 | 16.85 |  | Georgi et al. (2013) | CCCGATCGAAATAAGGAACA | ATTGACGACCCAGACTCCAC |
| Ig13662a | 6 | 19.74 |  | Georgi et al. (2013) | CATCTAGCCATGCACCATTG | CCAAGTTCGACATTTTCCGT |
| scf207d | 6 | 19.74 |  | Georgi et al. (2013) | GACACACGTGGTGCACTGTT | GGTTGATCTTAGGAGCTGCG |
| SCF13711 | 6 | 28.08 | KP278640 | Schlautman et al. (2015) | GACTTCCTTGGTACTTGGTG | ACTTTGAGGGTAGGAGTAAACA |
| ct188529 | 6 | 34.29 | KP279148 | Schlautman et al. (2015) | TTGCAGAATCAATAGTACCTCC | CCTCATTAGCTATGGTGAAAC |
| scaffold_63419 | 6 | 37.84 |  | Herein | TTTAGTCGTGTGGAGGAAAA | GACATTGAAGAGAGAGGAATTG |
| 1trimcontig344502 | 6 | 42.97 | KP279241 | Schlautman et al. (2015) | TGGAAATGGAAAAGTCTCTG | CACCGTCTACAGTTTAAGAACA |
| NA824 | 6 | 43.53 |  | Boches et al.(2005) | AAATCGTTGGTTTGGCTCTG | GTTTGGGCCGAAAAGAAATCGTAT |
| 314831_K70 | 6 | 43.82 | KP279219 | Schlautman et al. (2015) | ATCTCTCGTGCCTGTCATAC | CTTTTCGATGTCGTACTTGTC |
| vm53000 | 6 | 45.8 | JF834283 | Zhu et al. (2012) | CTCTCTCTAGCCAAGCAGATAC | AAGATGTGAGGAAGCTAGGAG |
| scf112c | 6 | 45.88 |  | Georgi et al. (2013) | ATGTGATTCGCGAAGGATTC | GAAATCCGGGGGTGTAAACT |
| SCF139334 | 6 | 47.23 | KP278874 | Schlautman et al. (2015) | GAGGGTCTAATATCTGGTTTCA | GAGAAAAGATGGAGCAAAAG |
| 412234_K63 | 6 | 49.7 | KP279182 | Schlautman et al. (2015) | GTGCAAGCCGTTTCTTATG | ATCGGAGGTTCCATCATTTA |
| SCF9909 | 6 | 49.79 | KP278630 | Schlautman et al. (2015) | CGTAGGTGGATTTCTCTACAAT | GGCATCTTATTTATCGTCTCTG |
| 1trimcontig238080 | 6 | 51.28 | KP279231 | Schlautman et al. (2015) | AGGGGTAATCTTCACACACTTA | ACAGGCTCTTCTAATCGTTTC |
| SCF3427 | 6 | 51.28 | KP278606 | Schlautman et al. (2015) | GCAAGACATCATCACAAACA | CTTATCCCAGTCCTTCAACTTA |
| SCF22442 | 6 | 53.07 | KP278657 | Schlautman et al. (2015) | ACAAAGAAAGACACTCCATCTC | GTATTTGACTTCCATGACCAC |
| vm27120 | 6 | 55.34 | JF834265 | Zhu et al. (2012) | AAGGTCTAAGAGTTATACCGCA | GGGCATAAGTTAAGAGAGCTAA |
| SCF92414 | 6 | 58.66 | KP278793 | Schlautman et al. (2015) | GTTATCCTCCCTTTGATATGTG | AAGAGCAACAAGATGGGTACT |
| SCF40517 | 6 | 66.18 | : | Schlautman et al. (2015) | GTAGAATGGCAATAGGGTTT | GAAGAAGATGACGAAGATCAC |
| 319429_K63 | 6 | 66.77 | KP279171 | Schlautman et al. (2015) | GGAGATAGGAAGTGTGATGAAC | TTATTGTGCAAGCATACGAG |
| SCF133376 | 6 | 68.59 | KP278865 | Schlautman et al. (2015) | ATTAGCACCGAATTTAACACC | GATTATGGGTGAGTCTGTGAAT |
| SCF89801 | 6 | 69.16 | KP278790 | Schlautman et al. (2015) | TAAACCTGTTCCGTCTCTTAGT | CTTTACTGTTGTGTTGTCTGCT |
| ct154615 | 6 | 70.35 | KP279138 | Schlautman et al. (2015) | AAAATTGAGCACTGGCTAAG | CTCATACAAACAATAGGGGG |
| scaffold_4374 | 6 | 70.35 |  | Herein | TCACTCAACACCAACACTAAAC | CATTGTTTTCCCTATCTCTCTC |
| scf17d | 6 | 70.35 |  | Georgi et al. (2013) | TCGCTTGAAGCTTACCGAAT | AGAACGAACACCTCGGTCAC |
| SCF31172 | 6 | 70.35 | KP278686 | Schlautman et al. (2015) | ACTGGATCTGGTGTTATTTACC | GGCTGGAAACAATTCAAAC |
| SCF16407 | 6 | 73.28 | KP278648 | Schlautman et al. (2015) | GGCAGTGAATTAAAGGTCAAC | GATGAGAAAGAAGAGTAAGGCA |
| SCF171621 | 6 | 74.47 | KP278919 | Schlautman et al. (2015) | CACCACTCCCCATTTTAAG | AAGGGACAGAGGAAGTATTTG |
| SCF89447 | 6 | 74.47 | KP278787 | Schlautman et al. (2015) | TAAATAAGACCTTCTGCTGACC | AATATGCTCACCACCAGTAAAG |
| SCF25446 | 6 | 79.54 | KP278665 | Schlautman et al. (2015) | TAGTGTGGACTTAACATGGAGA | ATCCAACCAAGTATCAGCAA |
| contig652 | 6 | 80.75 |  | Georgi et al. (2013) | AAAACTGTCGGCAGATCCTC | GGGATACCAATGTGGGTCAG |
| Pr031818823 | 6 | 83.12 |  | Blueberry Markers | AATCTCTGTCGCCCATTTTG | TTCCCCTGCTTCTGCTGTT |
| SCF164915 | 6 | 83.12 | KP278915 | Schlautman et al. (2015) | CTCAAAGTATCTCACTCACGC | ACTGTTGTCCCCTCTGACTAC |
| SCF54555 | 6 | 83.12 | KP279025 | Schlautman et al. (2015) | TTACCAAAGCACCCATTAAC | ACGACACATATCTCCAAAGTG |
| scf44a | 6 | 87.4 |  | Georgi et al. (2013) | ACAAAACCACTGGCGAAAAC | GAGTGACCAGGGGAGATGAA |
| SCF126708 | 6 | 87.61 | KP278854 | Schlautman et al. (2015) | CGACGAATAAACAAATCAAGTA | GAGAAGAAGTGAAGGAGAGTTG |
| 16720_K63 | 6 | 88.38 | KP279159 | Schlautman et al. (2015) | CTACCTTTCCCTCTCCTTGT | AGTTGAAGCTGAGAATTGTACC |
| ct139553 | 7 | 0 | KP279125 | Schlautman et al. (2015) | GATCAAGCATTGTTCTCTTCC | AGCTATAGGGCTAGCGATG |
| scf1594 | 7 | 0 |  | Georgi et al. (2013) | ATGCGAATGGAGAAATCTGG | ATACCGCAAATGGAGTCTGC |
| scf2s | 7 | 2.38 |  | Georgi et al. (2013) | TGAGACGTACGCACTAGCCA | GTCGATGGTGTTTGTCGATG |
| scaffold_11617 | 7 | 4.76 |  | Herein | TCTCTCTTCTCTCTCACTTTCC | TATCCGCTATCTCATCCTTTAG |
| scaffold_20967 | 7 | 4.76 |  | Herein | TTCGTTTTAGAGAGAGAGAAGG | GGAAGCAGTGAATATGGAGTAT |
| SCF56717 | 7 | 7.16 | KP278733 | Schlautman et al. (2015) | GTGTTTGTGTTTGTGTCTGTG | GATGATTTCACCTACATCGG |
| SCF2483 | 7 | 11.64 | KP278601 | Schlautman et al. (2015) | TTTCCTTCATAGTGTTGCCT | GTCTCCCTGTTAAATCCACTC |
| SCF25944 | 7 | 11.64 | KP278666 | Schlautman et al. (2015) | AACTATGCCAGAAGACTCAGAT | CTTCACAAATCACAACCACTAC |
| 1trimcontig435620 | 7 | 12.29 | KP279244 | Schlautman et al. (2015) | CAACCAGCCTTACAGTGAATA | GTCCGTTCAATTTCTTTTCC |
| 6ms4e4b | 7 | 15.75 |  | Georgi et al. (2013) | GGCCAAGGTTCTACCCTTTC | CAACTACCCACCACCACCAT |
| SCF20681 | 7 | 16.77 | KP278653 | Schlautman et al. (2015) | AGCCTAAACCTCTGTTTGATG | TTACAATACCTCGCTCCTTAGA |
| SCF36745 | 7 | 16.77 | KP278698 | Schlautman et al. (2015) | TCCTCATTAAGTATTGGACAGG | CTGGATTCTTGTTCTTAGCTTC |
| scf137c | 7 | 19.56 |  | Georgi et al. (2013) | CTCCGGGAACTCTCCATACA | CTTCGTTGTGAACGCAAAAG |
| SCF7155 | 7 | 19.78 | KP278617 | Schlautman et al. (2015) | GGGATCTATGAGTTGTGGACTA | CCACGGAATAGTTGTAAGTTGT |
| SCF89247 | 7 | 21.95 | KP278786 | Schlautman et al. (2015) | TGGAGGAGGTGAAGAATACTAA | CCCTTTGGACAACAAAATAC |
| scf31h | 7 | 24.33 |  | Georgi et al. (2013) | TGGAACTCCAAATGTGCGTA | TGGCACCATAAATAGCACGA |
| SCF25221 | 7 | 25.82 | KP278664 | Schlautman et al. (2015) | GTATCCCCACACTTACCACTAT | AGGATTGGACGGTAGCTTA |
| SCF85946 | 7 | 29.45 | KP278781 | Schlautman et al. (2015) | TGTGAACAGAACCTACCACTAA | AAAGAGCCCCGTAGATAGAT |
| SCF184873 | 7 | 32.42 | KP278934 | Schlautman et al. (2015) | AAGCGTAGAATATGTATGACCC | GGTAGTCCTCACGGAAGAG |
| SCF116864 | 7 | 32.73 | KP278836 | Schlautman et al. (2015) | TGCCCCTTGATTCTAATTTT | ATGCCTCAGATTGATTTACCT |
| SCF112540 | 7 | 34.8 | KP278830 | Schlautman et al. (2015) | CAGTAGTGGTATTTCACAATCG | TTTAATGCTTTTGGAAGAGG |
| SCF111145 | 7 | 35.19 | KP278827 | Schlautman et al. (2015) | TTAGTCTGGCTGGTTTTAGTTT | TTGTACCTATTGTTGGATTGTG |
| 80734_K70 | 7 | 41.83 | KP279198 | Schlautman et al. (2015) | AGGGAGAACCAATTCCTTAC | GACCTAACCCTAACCCAGTC |
| ct145906 | 7 | 41.83 | KP279132 | Schlautman et al. (2015) | TCTAGACTTGAGAAGCACTTTG | AGTTAGAGGAGGTTTCTGTTGA |
| SCF94237 | 7 | 41.83 | KP278795 | Schlautman et al. (2015) | ATCGCATCAGGTAAGCTAGTAT | TCGAGTGTCATTGTAATAGGC |
| 1trimcontig217158 | 7 | 44.8 | KP279229 | Schlautman et al. (2015) | GGAGTCGGTAAAATCAAGAA | CCAAATTCAGTAGGAGTACACA |
| 76326_K70 | 7 | 49.58 | KP279197 | Schlautman et al. (2015) | AATGTCTTCCAAATCAGGTG | CAAGAACGAACCCTCTATTTC |
| SCF34513 | 7 | 49.58 | KP278695 | Schlautman et al. (2015) | TACTAATCTTCTGGTTTGGGC | GTACACCACTCCTGATGGC |
| 409618_K63 | 7 | 50.01 | KP279178 | Schlautman et al. (2015) | CTTCTCCTTCCCTTCACTTTA | TTAGTGTTAGTGTTGGTGTTGG |
| SCF13006 | 7 | 50.21 | KP278639 | Schlautman et al. (2015) | AAAACATAAGAAAGAGCCCC | GGATGATGATGTATGGGAAT |
| SCF72379 | 7 | 51.93 | KP278757 | Schlautman et al. (2015) | TAAGGAGATCGACTAGGGTTT | CATCAAGATTCAAGACCACAC |
| SCF34010 | 7 | 52.2 | KP278693 | Schlautman et al. (2015) | GAGAATATGTGATGTTGAGGTG | CAAGTGTTAGGCTCGTTTAGTT |
| ct147864 | 7 | 52.4 | KP279133 | Schlautman et al. (2015) | CTCTCTTTACCCTCAATTTCTC | GGTCTAATATCAATCGATGACC |
| SCF137494 | 7 | 52.4 | KP278869 | Schlautman et al. (2015) | CCAACATAAAGAGGACTAGAGG | GACCTAGACTCCAAATCACG |
| ct145217 | 7 | 54.32 | KP279131 | Schlautman et al. (2015) | CCAGTACTAGATCCACTGCATA | TGTTCTAGAGAGGATGACATTG |
| SCF208883 | 7 | 54.35 | KP278952 | Schlautman et al. (2015) | GAGGAGTGAAGAGCCAGTAA | GACATTTCAAGTCCCACACT |
| SCF9872 | 7 | 54.35 | KP278629 | Schlautman et al. (2015) | ATGGGAGTGCATGAATAAAC | GGAGAATCGTATTTGTGAAGAG |
| SCF138014 | 7 | 56.06 | KP278870 | Schlautman et al. (2015) | TTATTCTCTTCGCTTGGGTA | TCAGATCATGGATTACTGGTT |
| 300409_K63 | 7 | 56.13 | KP279168 | Schlautman et al. (2015) | GGGGAATAGCAGGTAGTGAT | TATTTATCCACCCACTTCACAG |
| 1trimcontig337780 | 7 | 59 | KP279239 | Schlautman et al. (2015) | CTTGATCTTGTCGCTGTAGAC | CTGAGCATCTCTCCTTTATCTC |
| 311291_K70 | 7 | 59 | KP279214 | Schlautman et al. (2015) | CTTGATCTTGTCGCTGTAGAC | TTCCTTATCGAAATCACGAG |
| SCF4305 | 7 | 59 | KP278611 | Schlautman et al. (2015) | AATGAGTGGTTATGTAGGGAGA | AGATTGGTGAGATATGAGGAAG |
| SCF128015 | 7 | 62.02 | KP278856 | Schlautman et al. (2015) | ACCCACTCTTTCTATTATCTTCC | GTGAGTTCCAAGTTCCACATA |
| NA1792 | 7 | 64.37 |  | Georgi et al. (2013) | GCATCATCGCCGTCAAG | TTGACTTCATCGAAAGCACG |
| ct95345 | 7 | 64.69 | KP279106 | Schlautman et al. (2015) | ACTCTACAAGGGCACGAAC | ATGGAAGTAAGAAAGTGAGTGG |
| SCF128307 | 7 | 65.87 | KP278857 | Schlautman et al. (2015) | ACTCAGAAGTTGAAGCACAAA | GTATCAAGTACACCAACACCAG |
| SCF193103 | 7 | 67.35 | KP278943 | Schlautman et al. (2015) | GAGGAGTTGAAACAATTAGTCC | TACCCACTTTAGTCGAAGGAT |
| SCF110168 | 7 | 68.54 | KP278822 | Schlautman et al. (2015) | AAAGGACTAGAGGGAAGTACAAC | CTTATTATCCAGAAACTCGTGC |
| scf5k | 7 | 70.33 |  | Georgi et al. (2013) | GCATTACTAACAGCATCCCAA | GAGCCACTTTTCACTCCCAA |
| SCF187979 | 7 | 76.89 | KP278935 | Schlautman et al. (2015) | AGATAAGGCACCCGATAATAC | GATCAAGGAACGCAAATCT |
| 419834_K63 | 7 | 77.73 | KP279192 | Schlautman et al. (2015) | GAAAAGAGAGGAGAAGATGGAT | TACCAGAACTGTGTGAGATTGT |
| SCF167793 | 7 | 79.27 | KP278916 | Schlautman et al. (2015) | GTGAAACGACAAGACCAAAT | AGGACATCCACCTTCAAAT |
| SCF915 | 7 | 80.46 | KP278593 | Schlautman et al. (2015) | TTAGGGTTTGGAGTACCTGA | ACTACCGTCTTTCTTTATAGCC |
| Ig51a | 7 | 85.62 |  | Georgi et al. (2013) | TTGGTGCAAGATCACCACAT | GCACAAACGGATGTAGCAGA |
| SCF46824 | 7 | 89.89 | KP278717 | Schlautman et al. (2015) | GGAGATGCTGTAATAACGAAGT | TTAGTCAATATGCGTGCAAC |
| SCF10514 | 7 | 90.38 | KP278632 | Schlautman et al. (2015) | GTACTCTTTGTCGGATGTTTTC | GTTTCACTCCCACCTCTTAAT |
| SCF155637 | 7 | 90.38 | KP278901 | Schlautman et al. (2015) | TGTTAGTGTTAGGACCCGTTA | AAAGTAGGAGTTAGGATGGGAT |
| 1trimcontig450309 | 7 | 90.7 | KP279253 | Schlautman et al. (2015) | AAAATCAGAGGGAAGAAAGC | TATTAGCCAGTCCTCCTTTGTA |
| scf203h | 7 | 95.5 |  | Georgi et al. (2013) | AAGTTACAACGGTTCGTGGC | TGCAACATTGTGATGGTCCT |
| ct121951 | 8 | 0 | KP279117 | Schlautman et al. (2015) | CATGTAGCCGACTCCAATTA | TATCCCATTCCGTATAAGGTC |
| 2ms2g09 | 8 | 1.78 |  | Georgi et al. (2013) | GGGGAACTCAGATGGGTTTT | GCTGTCATTTTTCGGAGAGC |
| scf1p | 8 | 1.78 |  | Georgi et al. (2013) | AGAGTTGCCTCGAAGTAGCG | TGGGTGTGCTGAG |
| 1trimcontig440337 | 8 | 1.79 | KP279250 | Schlautman et al. (2015) | CTTGGAGTTAGCCTTTTAGTCA | CTGGAAGAGTGAAGATGGAATA |
| SCF10785 | 8 | 1.79 | KP278633 | Schlautman et al. (2015) | ACATAAAGGAGAGGGAGTAGAG | ATACCACTTGATAGATTCCTCC |
| vm28527 | 8 | 1.79 | JF834266 | Zhu et al. (2012) | GGACAAGTGAAATGCTAGTTG | AGATTGTTCGTAGGTAGAAGTG |
| KAN-11325 | 8 | 2.38 |  | Blueberry Markers | CAACATTCCCGAAAACCAGT | ACCCTTCACCTGACACCATC |
| SCF102538 | 8 | 2.38 | KP279056 | Schlautman et al. (2015) | TTACTGGGCAATAGAAGGACT | CACATAAGTTTGGCTACACAAC |
| vm31502 | 8 | 5.31 | JF834267 | Zhu et al. (2012) | TTCTTTTGTCCACCTTGAGT | TCTCTTCACTTATTACACCTGC |
| 1trimcontig326802 | 8 | 6.5 | KP279235 | Schlautman et al. (2015) | TTTTCAGAGCAAGAGGAAAG | CTGTCTGTATCATGGAACTCAT |
| SCF91821 | 8 | 6.5 | KP278792 | Schlautman et al. (2015) | TTCTGTGTCTGATTCCATCTC | ACTAGCCCAACAACTTAGACTG |
| scf2505a | 8 | 10.91 |  | Georgi et al. (2013) | CCAGAGAGAAGGGGGAAATC | TTATCCCGCCGCTTAGTAGA |
| SCF107477 | 8 | 14.65 | KP278814 | Schlautman et al. (2015) | GTCTTATTTTCACTGTCGTGTG | CGGGCATTAACCTTATACCT |
| scaffold_37046 | 8 | 17.03 |  | Herein | AACACATCTCTTATTACTCGCC | CCTCCTCTCTTGAAAACATCT |
| scaffold_54259 | 8 | 17.03 |  | Herein | ATAAGTTGGGCTAGTAAAGGG | GATGGTCCCCTAAGAATATAGA |
| SCF71136 | 8 | 17.46 | KP278753 | Schlautman et al. (2015) | TCTGTTTTCACAGCTATCACAC | GTTCATCAAAGGCCAGAGT |
| 36394_K70 | 8 | 20.98 | KP279271 | Schlautman et al. (2015) | CAGTGTTTGTTGCTTGGTC | ATCTCACTCTCTGTTTCCCTC |
| SCF8850 | 8 | 25.19 | KP278623 | Schlautman et al. (2015) | GTGTGATGTATTTAAGGAGTACCAC | ACAGATAGAGTAGTTACCAAGGGA |
| SCF92564 | 8 | 28.75 | KP278794 | Schlautman et al. (2015) | TCATAACTCCCTCGTAATCAAG | AGGAAGAAGAGAATAAGGTTGG |
| SCF77645 | 8 | 36.23 |  | Herein | GGTTCTTTCTTCTGGGTTTT | TCAGACAATGAGCTACTACCCT |
| 418192_K63 | 8 | 39.74 | KP279266 | Schlautman et al. (2015) | CAGGCAGAAGAAGAAAGAAA | TGAATTAAGAGAGGAGGAGAGA |
| 260167_K70 | 8 | 41.09 |  | Herein | TCAACATCTTTGGGACTTCT | GCTTGCCTAATATACTTCCAAC |
| 314797_K70 | 8 | 41.09 | KP279218 | Schlautman et al. (2015) | CTTGTTCTCCTCCTTTAGTCTG | CATCTTCATACTCCTATTGTCG |
| SCF27811 | 8 | 41.09 | KP278672 | Schlautman et al. (2015) | ATGTGACTAGCATGGGACTTA | TATTTACCTGGATAGGAGAAGG |
| SCF71184 | 8 | 43.47 | KP278754 | Schlautman et al. (2015) | TCTGTTCAGTTGGGCTTTAT | GCTCACATTCACCTGTAATTC |
| 187382_K70 | 8 | 46.38 | KP279202 | Schlautman et al. (2015) | CCTCCATTCTCTCTCCTACTAA | CTTCTTCTCTCTCTC |
| ct159707 | 8 | 51.74 | KP279142 | Schlautman et al. (2015) | TGTTAGCTCCTTACTTTCCATC | GTGAAGAGGAAGATGAAGAATG |
| scaffold_50168 | 8 | 51.74 |  | Herein | CGTTCCAAAATAAGCGTCT | CATCTGCCTAATATAACTGGGT |
| scaffold_48237 | 8 | 51.81 |  | Herein | CTCTCTGCTGTTTTCATCAAC | GCTATTAAGGAAGGGTCAAAC |
| SCF197903 | 8 | 51.81 | KP278945 | Schlautman et al. (2015) | TCTCGTGAGCGTTACAATATAC | ATGGAGTCAAGGTAAACCG |
| ct134336 | 8 | 52.33 | KP279123 | Schlautman et al. (2015) | GAACACTCCTTCTCTAGCTCTG | CTTTTTAGTCTCCGACAATCTC |
| SCF142767 | 8 | 52.33 |  | Herein | ATAGTTGGACGGGTGTAATG | CTCTCGCAAAGTAGAACAATCT |
| SCF19565 | 8 | 52.33 | KP278652 | Schlautman et al. (2015) | GGGTTTTATGAGTTAGAGTCCC | GTAGCGATGGTCTT |
| scf4860 | 8 | 52.93 |  | Georgi et al. (2013) | TTCGCTCAAGTCAACTGTGG | CCTTGGACATTTTTCTGGGA |
| SCF64632 | 8 | 52.93 | KP278746 | Schlautman et al. (2015) | ACCTCCTAAAACACAACCCTA | CTGAGTAATCTTCGATGTGAGA |
| SCF82870 | 8 | 52.93 | KP278775 | Schlautman et al. (2015) | GCTAAAGAACGAACAACAACAC | GTCCAACGAGTGAGTAGAGAAG |
| SCF127023 | 8 | 54.64 | KP278855 | Schlautman et al. (2015) | TATGCTAATCCACTTTGTAGGG | AATCTGGGTAATTGGGAACT |
| SCF54155 | 8 | 54.64 | KP278728 | Schlautman et al. (2015) | TCGAAGAAAATGAAGGGAC | ACAAATGGAGAGGAAAGTGTAG |
| SCF87990 | 8 | 58.84 | KP278783 | Schlautman et al. (2015) | GTGTAGGTGTAAATGTGCTTTG | GGCGTATAAAAGGATTCAAG |
| SCF30010 | 8 | 58.86 | KP278683 | Schlautman et al. (2015) | CTCAAATCAACGATCAAGAC | GAAAGAGACAACAAAACCCT |
| SCF2714 | 8 | 60.93 | KP278602 | Schlautman et al. (2015) | ACAAGTCTCTGGAAGCTAACAT | GTTGATTGTTGGGTCTAAGTTC |
| SCF189612 | 8 | 62.21 | KP278936 | Schlautman et al. (2015) | GAGGATTGTTAATGGTTTCTTT | TACGCTTCATCTTGTTATTTTC |
| Pr031818828 | 8 | 64.22 |  | Blueberry Markers | TCGTTCTATTCCTCCCGAAT | ATAGAAACTCGCCGTCTCCT |
| vm13884 | 8 | 64.22 |  | Zhu et al. (2012) | TAAAGCTATGTATGAGCCGATG | GTTTTGGCAAATAGACTATCCC |
| SCF17979 | 8 | 66 | KP278649 | Schlautman et al. (2015) | ATATCAGAACAAGGAGATGGTG | GATACCGAATGAACCAAGAA |
| 411348_K63 | 8 | 69.16 | KP279180 | Schlautman et al. (2015) | AATTACCAATGTTCACTCCG | GTTGATGTAGTTCTGTGGTTGA |
| SCF105925 | 8 | 70.35 | KP278812 | Schlautman et al. (2015) | CCGTGTCAAAAGATCAAGC | AGTTTGTGCCGTCGTACTC |
| SCF172149 | 8 | 76.65 | KP278922 | Schlautman et al. (2015) | GTTAAATGATGCTGTTAGGGAG | ATGTCCAGTCGTTATCTCTAGG |
| SCF81732 | 8 | 80.22 | KP278772 | Schlautman et al. (2015) | CGAGTATGTGGAGAGGCTTAC | GTGTATAAAATGGGCATCACAC |
| SCF132922 | 8 | 81.37 | KP278864 | Schlautman et al. (2015) | TTAGACGCTTTATGTCCATTC | GAGTGTCCTTGTCTTTGTTGTA |
| ct89379 | 8 | 84.69 | KP279101 | Schlautman et al. (2015) | ATGAAGAGCTTGAATGGCTA | ACACTTTACACCACAACTCGTA |
| Ig6523b | 8 | 84.69 |  | Georgi et al. (2013) | CCATCTACCACGGCAGAGAT | GCATATTTTGGTTGGATCGG |
| SCF138992 | 8 | 84.69 | KP278873 | Schlautman et al. (2015) | ATACTTTACCCCACAGAGCTTA | CCACTCATGCTCACATCAC |
| SCF24087 | 8 | 84.69 | KP278663 | Schlautman et al. (2015) | GTCCCTTTCTCGTGTCTTTAT | GAGTAGTGACGATGCAACTAGA |
| 1trimcontig328266 | 9 | 1.13 | KP279236 | Schlautman et al. (2015) | ACAGATCAAGCGAACACTAAAC | CCTGCTCCTGTTATACTACCAA |
| SCF3932 | 9 | 1.13 | KP278610 | Schlautman et al. (2015) | CAGAGTTTCAGTGGAGCATT | CTCAGCTTCTGTGTTTTGTGT |
| scf45d | 9 | 1.13 |  | Georgi et al. (2013) | TTCTTGTGGTTGTGCTGCAT | TAATGGCTGAAACGCTCACA |
| SCF125251 | 9 | 3.37 | KP278851 | Schlautman et al. (2015) | TATACAGTCAGATCCAATCCAC | TGCAGATAAAGTACAAGAGTGC |
| SCF61189 | 9 | 3.37 | KP278744 | Schlautman et al. (2015) | GCCATAACTCTCACTCAAATCT | ACCTATTCACCTACATCCAAAG |
| scf55c | 9 | 4.55 |  | Georgi et al. (2013) | AGCCATTGATCTCCAACCAC | GCGTTTCAATCTTTGGCAAT |
| 1trimcontig439861 | 9 | 7.53 | KP279247 | Schlautman et al. (2015) | CTCCTCTCTCGAATGACACTAC | TTCTTGTTGGCTGGAGATTA |
| SCF132532 | 9 | 7.53 | KP278861 | Schlautman et al. (2015) | GACTGGATTTTCACGAATCTAC | CTTCATCTTCCTTGACACTTCT |
| scf32j | 9 | 8.81 |  | Georgi et al. (2013) | ATCCACCAAACAAGCCACAT | TCAATCAACGCGATTCCATA |
| SCF11802 | 9 | 11.64 | KP278638 | Schlautman et al. (2015) | CGAGGAACAAGTTTTATAGGAG | ACACTCACCTTTATTATGGGAC |
| SCF33185 | 9 | 11.64 | KP278691 | Schlautman et al. (2015) | AGCACACTACAGACAGGGTAAT | GTTTTGGCTCTGGCTAAGTAT |
| SCF132369 | 9 | 12.24 | KP278860 | Schlautman et al. (2015) | CTACTTTGGGATGGAGAGAGTA | AGGTTTAGGTAGTGTTGGATTG |
| SCF136317 | 9 | 12.24 | KP278867 | Schlautman et al. (2015) | GAGAGTTCAAATTACCTGTACCA | GGAGATTAGGTTGTGGACTAGA |
| SCF144748 | 9 | 12.24 | KP278884 | Schlautman et al. (2015) | ATTTCCAATCCTTTCCTCTC | CTCTGACACCTTCTGACACATA |
| scf306f | 9 | 13.31 |  | Georgi et al. (2013) | GGGCAAGGATAAAGGGTTGT | TGCATGCAACTTCCTAGTCCT |
| ct95842 | 9 | 13.66 | KP279107 | Schlautman et al. (2015) | GTGGAAAGAGATTGTTGATGTC | AAAACTAATGGATGACGACG |
| 1trimcontig238343 | 9 | 21.73 | KP279232 | Schlautman et al. (2015) | GGTAATAGCTTTGTGATCTTGC | GATGGTGAATAAATTGCGAC |
| 214102_K63 | 9 | 21.73 | KP279165 | Schlautman et al. (2015) | GGTAATAGCTTTGTGATCTTGC | GGGTAAAATGACTGCCAAC |
| SCF141985 | 9 | 21.73 | KP278878 | Schlautman et al. (2015) | GAATGGTCTTGAGGGATGTAT | ACTCTGGAAGAAATAAAACGG |
| SCF1648 | 9 | 25.15 | KP278599 | Schlautman et al. (2015) | GTTGATCTGAAGGAAACCAA | TCGTATTAACTCCCCTATTGAC |
| SCF88396 | 9 | 30.92 | KP278784 | Schlautman et al. (2015) | ATAGAGGTTAATTGGTCCTCG | GACGAAGAACGACAGGTAGAT |
| scf21n | 9 | 31.29 |  | Georgi et al. (2013) | ACCAATTCCCTCCCAAGTTC | CCCTGGATATTTGCTTGCAT |
| Pr031818814 | 9 | 32.8 |  | Blueberry Markers | CTCACCCATCCTTCTCCTCT | CGGTGTTGATGTCATGCTT |
| Ig1296a | 9 | 37.47 |  | Georgi et al. (2013) | CCCCTGAATTCTTGTTCCAA | GAGTGGAAAACGCAGTGGAT |
| SCF136207 | 9 | 37.47 | KP278866 | Schlautman et al. (2015) | GTCTCTGTAGTCGGTGCTTT | GATTTCGATTCCTTGACACT |
| SCF48414 | 9 | 37.47 | KP278721 | Schlautman et al. (2015) | GTAGGGAAACAAGAATTGGAC | ACTGTGAGATTGGTGTGATATG |
| SCF56032 | 9 | 38.06 | KP278731 | Schlautman et al. (2015) | AGAAATGGCGCTCTGTATC | GAACAGTCTCATCTTCACGAC |
| scf6i | 9 | 38.06 |  | Georgi et al. (2013) | TTGTTTGGTGCTACGAGTGC | GGCCTGAACTTTCCTGACTG |
| SCF118468 | 9 | 44.63 | KP278840 | Schlautman et al. (2015) | ATAAGCGGAGCACAGTTACA | GATAGGATGACCTGTTTTGGT |
| SCF10459 | 9 | 47.18 | KP278631 | Schlautman et al. (2015) | TCTTTGTTTCTGAGGTTGCT | ATTTGTAGGTACTATGGAAGCC |
| scf275d | 9 | 47.18 |  | Georgi et al. (2013) | GCTTTTCTGAAGCGATTTGC | CCGCATACACGGCGTACTA |
| scf13a | 9 | 56.29 |  | Georgi et al. (2013) | TAGAGGGCGTTGAAAGGAGA | CCCCAAATTTCTCCCCATTA |
| 309124_K70 | 9 | 56.79 | KP279213 | Schlautman et al. (2015) | AAAGGTCGTTAAGGCTATCAG | TGATGACTGCGATATGTACTCT |
| scf35k | 9 | 57.18 |  | Georgi et al. (2013) | TCACCTTAAACCCTGGCTTG | GTGGAGATGGATAGCTTGGG |
| NA619 | 9 | 59.09 |  | Georgi et al. (2013) | TCACACTACAGGCAGGAGAGA | GAAGCCCCAGTTCTCACAAG |
| scf439 | 9 | 62.74 |  | Georgi et al. (2013) | TTGTGTGATCCGCTACTTGG | ATCGTTCAAAACGAAGGGTG |
| SCF7357 | 9 | 62.74 | KP278618 | Schlautman et al. (2015) | CAGCTTAATCATCAGTTCCAG | AGTGAGCATCGACTATTTACCT |
| SCF155797 | 9 | 71.69 | KP278902 | Schlautman et al. (2015) | ATCATTAAGGCTCCCAAAG | GTACGTCTACTCTGACGGCTA |
| SCF30734 | 9 | 75.17 | KP278684 | Schlautman et al. (2015) | GTTGAAAACCCAACTGTGAG | AGATCCAGTCATGGTACTTTTG |
| VCC_J9 | 9 | 75.26 |  | Boches et al.(2005) | GCGAAGAACTTCCGTCAAAA | GTGAGGGCACAAAGCTCTC |
| ct154206 | 9 | 80.04 | KP279137 | Schlautman et al. (2015) | GAGAGCGTACGATACCTAATTC | CTGGTTAGGAAAACCACTAGAA |
| 1trimcontig352078 | 9 | 87.09 | KP279243 | Schlautman et al. (2015) | CGTGTTTCTGTTAGATAGCTTG | CTTGTACGTGAAGATGCAAA |
| 407841_K63 | 9 | 87.09 | KP279175 | Schlautman et al. (2015) | TTGAGTAGATACATGCTGGCT | CTCACCCTTTCTCTTGTGATA |
| ct144558 | 9 | 87.09 | KP279128 | Schlautman et al. (2015) | TCATTACCCCTAACCTCTAAAC | ATTCGACTAGAGTGGAGAGAAA |
| KAN-11281 | 9 | 87.09 |  | Blueberry Markers | GGGGTAACATTGACCATTCG | AAATCCCTCAATCCAAAGGG |
| SCF109660 | 9 | 87.09 | KP278821 | Schlautman et al. (2015) | CCCCAAACTGTCGTATAAAA | TAGAGTACAGGAAAAGCCCTAA |
| SIZ1 | 9 | 87.09 |  | Blueberry Markers | ATTGCAATCTTGCACAGAGAGA | CTACATAGGATACGCATTGGCA |
| ct154654 | 9 | 87.34 | KP279139 | Herein | GATTTCTAGTGGGAAATGAAGG | GGTGTATGTGTGTGATTAAGGA |
| SCF191642 | 9 | 87.38 | KP278939 | Schlautman et al. (2015) | CTACATCCACTAAATATCAAGGC | GATCAAGCCAAAGGAAGAA |
| SCF125889 | 9 | 87.94 | KP278853 | Schlautman et al. (2015) | TCTCGTGTATTTTGGAGTGA | GTTGTATCCTTTGTCGATTCT |
| SCF163134 | 9 | 88.51 | KP278914 | Schlautman et al. (2015) | CAGTGCAATTAGTTTCCTATCC | TTCTTGGGTTGGTTATTCAG |
| scf1655c | 9 | 88.51 |  | Georgi et al. (2013) | CATCTATTGATCAGCCGCAA | ACGACCATATGAGCCGAGTT |
| SCF41971 | 9 | 88.51 | KP278709 | Schlautman et al. (2015) | ATACTTGACCTCTATGGCTTGA | GTACTTACGTGTTTGGTTCGTT |
| contig259Fb | 9 | 89.86 |  | Georgi et al. (2013) | TTGCTGAAGCCCTAAGCAGT | AAACCAGATCTGTTGGACGC |
| SCF22993 | 10 | 0 | KP278994 | Schlautman et al. (2015) | GACTGTGCGTAGACTTGATCT | AAGTATGTGTAGGCCGAAAA |
| ct93137 | 10 | 2.96 | KP279104 | Schlautman et al. (2015) | AAGATTTCCGCTACAGTACCT | GCTATGGGTGTCTCAAAAAG |
| SCF56747 | 10 | 2.96 | KP278734 | Schlautman et al. (2015) | TTAGAGAAAGGTCCCAACAG | GAAGAGGCTAAGAGGTCATGT |
| 364103_K63 | 10 | 2.99 | KP279173 | Schlautman et al. (2015) | TACAAACCCTAAGCTCTAAACC | CGACTTGAGTGATACCAAAGA |
| ct161908 | 10 | 2.99 | KP279144 | Schlautman et al. (2015) | CCTAGGAGATGGGTCAAGAT | ACCACTGTCTTCCATATTCACT |
| SCF105151 | 10 | 2.99 | KP278811 | Schlautman et al. (2015) | CAGAATAAGATTGGGTAGAAGG | TTTGAGAATTACTTGGCACC |
| SCF120352 | 10 | 2.99 | KP278843 | Schlautman et al. (2015) | AGTTCTATGACCCCTAACTGAA | GAAAGGAAAGAAGCACTATCAC |
| SCF46833 | 10 | 2.99 | KP278718 | Schlautman et al. (2015) | GGACCGCCGTATTTAGTTA | GCCCATACCCCTAGTTATTG |
| SCF23210 | 10 | 3.59 | KP278660 | Schlautman et al. (2015) | TTGATACTCTCGACCTCTTCTT | GTGGTGTTCGACATGATTTAC |
| SCF26014 | 10 | 4.18 | KP278667 | Schlautman et al. (2015) | GGTCCCAGAATCAATGTCTA | GAAATCAGAGAAGAAACAGGTC |
| SCF60761 | 10 | 4.18 | KP278742 | Schlautman et al. (2015) | ACTTAAACATCGGTCCATAGAG | AGAGTCGTGTCCTTTCTTTTC |
| SCF96311 | 10 | 4.18 | KP278799 | Schlautman et al. (2015) | TGTATAATCTCAGGGGCATT | TTTCTCATTTCCTTCCCAC |
| SCF2288 | 10 | 9.55 | KP278600 | Schlautman et al. (2015) | CAATAGTAGTTTCGAGCTTTCC | GTTTCCAATTCAAGCCTCTA |
| SCF86438 | 10 | 14.96 | KP278782 | Schlautman et al. (2015) | CTATTGAAAACAAGGAACGG | CCTATACAACCTCTTCGGATAA |
| Pr031818817 | 10 | 15.56 |  | Blueberry Markers | CGTATTTAGGGATGGAGGGAGT | CGAGGACATCATCTGGCTCT |
| SCF139660 | 10 | 16.15 | KP278875 | Schlautman et al. (2015) | ATAAATCTACGTCCATACAGCC | GAGTACATACAAATCCTCTTTCG |
| SCF189657 | 10 | 19.72 | KP278937 | Schlautman et al. (2015) | CATCCTTGAAAATAGACAGACC | CTTAGAAGACCGCACTGAGA |
| SCF104688 | 10 | 21.21 | KP278809 | Schlautman et al. (2015) | ACAAAGAAATGTATGGCACC | CTTTTCGTCTCCTCTAATTCC |
| scaffold_84092 | 10 | 28.27 |  | Herein | CACAAACAGGCAGATTACTTTC | AACCGAAAACGAGAAATACC |
| SCF172906 | 10 | 31.73 | KP278923 | Schlautman et al. (2015) | CTGTTCAAGGATTTGTACTGG | TATTGACATGAGAAGCACGA |
| ct135942 | 10 | 31.85 | KP279124 | Schlautman et al. (2015) | CTACTTGCCTTCCTCTTTGAC | TAAATAATCCGTCCACGAAC |
| SCF112295 | 10 | 34.87 | KP278829 | Schlautman et al. (2015) | AACATCTCTACCTCTCACGTTT | TAGTATTAGTTGATTTGGCGTG |
| 409500_K63 | 10 | 35.42 | KP279177 | Schlautman et al. (2015) | GATTCCTGGGTGTAGTTCTGT | CTTAGTCTTTAATGCTGGCTCA |
| vm25796 | 10 | 35.42 | JF834263 | Zhu et al. (2012) | CACTTACCTGAATCCTCTTAGC | TAGAGGAGCCAAACTGATAACT |
| SCF7569 | 10 | 37.25 | KP278619 | Schlautman et al. (2015) | CCCAATAACGACTCATATACCT | ACCCAGTCAAAATCTCCTTT |
| 313928_K70 | 10 | 38.44 | KP279216 | Schlautman et al. (2015) | CAATTATCAAGGAGGCAATC | TCACAAATGAGGATCTACACAC |
| SCF146740 | 10 | 39.03 | KP278887 | Schlautman et al. (2015) | ATGGGACTGCTTATTGAACAC | CAAGTGGTGCATTGTGAGA |
| SCF147295 | 10 | 39.03 | KP278889 | Schlautman et al. (2015) | ACTGAGGTAAAAGAGGAGTACG | CCATCAAGGTCTCAATCTGT |
| SCF35507 | 10 | 39.03 | KP278697 | Schlautman et al. (2015) | GTCTAATCTAATGCAGAATGCC | AATGTGGACAACGAGTACATCT |
| SCF36905 | 10 | 44.43 | KP279011 | Schlautman et al. (2015) | GATAAGCTGTGCTGAAACATC | CGATAGGGGATAGAATTAGTCA |
| vm72062 | 10 | 44.99 | JF834244 | Zhu et al. (2012) | CACCCATAAGAGATAGAACAAG | CTATCAATCATGATCTTCAGCC |
| 71002_K63 | 10 | 45.75 | KP279161 | Schlautman et al. (2015) | CTTCAATCCACGAATACCAC | CAATTATGCAAAGGAGGAAG |
| SCF107715 | 10 | 45.75 | KP278816 | Schlautman et al. (2015) | AAAGCGAGTCAGAAACATAGAC | CCTATCAGTTCCTTTCCTATTG |
| SCF189827 | 10 | 45.75 | KP278938 | Schlautman et al. (2015) | TTCATTTCCTTACACTTCCC | GTTAGCTTCTTCTCCTTCTTCA |
| SCF82535 | 10 | 47.54 | KP278774 | Schlautman et al. (2015) | TAGAAGAGGAAAACTGACGGA | TTGATGCAATCTGACAACG |
| scaffold_21777 | 10 | 50 |  | Herein | GATGCTCTCTCTTTCAATTAGG | TTTAGGTCTTGGGTAGCACTAT |
| SCF65999 | 10 | 50 | KP278748 | Schlautman et al. (2015) | AGGTAGCATTAGACACGAGATT | GAGGTTTTACATGACCATTACC |
| SCF80520 | 10 | 54.17 | KP278769 | Herein | TAAAGTGTTTTGGACGGCT | GCACAAATTATCGGAATCG |
| SCF174394 | 10 | 61.46 | KP278925 | Schlautman et al. (2015) | GGTGGATGGAATGCTAAATA | CTTTATTGGTAGTGGATTGGAC |
| SCF49656 | 10 | 61.46 | KP278724 | Schlautman et al. (2015) | ACTCTTACCCTTGAAACCAACT | TAGGTGCATGAGACTTTTAACC |
| SCF150919 | 10 | 66.9 | KP278896 | Schlautman et al. (2015) | TTGTTAGCACTTAGCATAACCC | GCTTCATCTCCACCAATACAT |
| 1trimcontig176861 | 10 | 71.39 | KP279223 | Schlautman et al. (2015) | ATGGATGTATCTTGACAGGC | CTGCTGTTCATTTCTCTGTG |
| 60699_K70 | 10 | 71.39 | KP279196 | Schlautman et al. (2015) | CTTCTCACTGTATTTCTTCGAG | GGCTACTTTGTTAGGGTAGATT |
| 418931_1_K63 | 10 | 72.58 | KP279191 | Schlautman et al. (2015) | ATTAGCTCAGTTCCCAGTAACA | CTTCTTTCTCTTTCTCCTTCCT |
| scaffold_11771 | 10 | 72.58 |  | Herein | GAGATCAGAGAGGAGTAGTTGG | ATAAGGGAGAGTTGGTATAGGA |
| scaffold_71386 | 10 | 72.58 |  | Herein | GAAGTAGCTGGACTGATGTATTC | CTCTCTCTTCCCCTTTTACTCT |
| vm38401 | 10 | 77.28 | JF834272 | Zhu et al. (2012) | CAATGGGAAGTACAAAGAGC | CGATGCAATCTTAGTCTTGA |
| SCF201915 | 10 | 79.07 | KP278947 | Schlautman et al. (2015) | ATGCACATCCTGAAGTACCA | CTGAACACATTGGACGGAT |
| vm12486 | 10 | 79.07 | JF834257 | Zhu et al. (2012) | GGTGGAGATGCTCGTAGTATT | CTAAGGGACGTCAAACCTAAC |
| ct145170 | 10 | 79.75 | KP279130 | Schlautman et al. (2015) | GAATCCTAGCCTATTTCCTTTG | GAAGCAAACACCACTCAATATC |
| KAN-11049 | 10 | 79.75 |  | Blueberry Markers | CTGGCTCTGTAGACCTTGCC | AACGGATTATACTGCCACGC |
| SCF84804 | 11 | 0 | KP278779 | Schlautman et al. (2015) | CTAGTCTTCTTGTGACCTAGCC | TATTCTTTTAGTCCGAGCCA |
| SCF110507 | 11 | 1.19 | KP278824 | Schlautman et al. (2015) | GTAGCTGAGGTGGAGGATAAC | GAGCTGGTGCTGAAATTAAC |
| SCF181909 | 11 | 1.19 | KP278932 | Schlautman et al. (2015) | CTCTCAATCTCTTGTTTTCTCC | TTCAAACCTCAGCAATCAG |
| SCF72209 | 11 | 1.19 | KP278755 | Schlautman et al. (2015) | CTTTACCTTTTCCTTCAGTCGT | GAGGTTCACCAAATCTTACCA |
| SCF95767 | 11 | 1.19 | KP278796 | Schlautman et al. (2015) | TGAGGAGAGGAGTATCCATAAG | CCTACAAGTCTCGCAATTCTA |
| scf108b | 11 | 2.38 |  | Georgi et al. (2013) | ACATAAACGGCGATTCCAAC | ATTGCTCGAGGATTGGACAC |
| 1trimcontig176042 | 11 | 4.39 | KP279221 | Schlautman et al. (2015) | CCGTTGTTGTTCTTCTGTAGT | TTCAACCTCTGAAGCCTCT |
| vm05418 | 11 | 6.87 | JF834252 | Zhu et al. (2012) | GGGATAAACACTTACAGGAAGA | CTAGCTAGCCGTCAGTTATTTC |
| SCF41361 | 11 | 8.3 | KP278708 | Schlautman et al. (2015) | AAAATTGCTTGGTCCTCAC | AAGTGTATAGTCTGGGGTGTTC |
| SCF122746 | 11 | 11.07 | KP278846 | Schlautman et al. (2015) | ATTGTATGAAAACCCTAACCC | GAGACGATTCCAAATATAGCA |
| 307018_K70 | 11 | 14.05 | KP279208 | Schlautman et al. (2015) | TAAAACCTTACCTCCTCTTCTG | TAACCTCGGATCTCCTTATCTA |
| SCF81909 | 11 | 14.62 | KP278773 | Schlautman et al. (2015) | TAGAGGAATCAGCAACTTCACT | TTCACACTCACACTCACACG |
| SCF204332 | 11 | 23.86 | KP278948 | Schlautman et al. (2015) | CGTGATCTCCCAGAGTTGT | CTTTTATTTCCCTATGTGTCCC |
| Pr031818821 | 11 | 26.07 |  | Blueberry Markers | TCTAGGGTTTTGGCGCTTC | TCCTTGAGAACAAGTACAGGTGAG |
| scf3072b | 11 | 26.67 |  | Georgi et al. (2013) | AGTTTAAGCGGAGCGAATGA | TTTGGCGACATTTTTCTTCC |
| SCF81294 | 11 | 26.67 | KP278771 | Schlautman et al. (2015) | CTATCGACGGCTGAGATTT | AAAAGGGGAAGATCCTAGAAG |
| SCF106182 | 11 | 36.88 | KP278813 | Schlautman et al. (2015) | TACCCTTGTGTATCCCTACATT | GAACAATAGCAGCAACAGAAC |
| SCF199831 | 11 | 36.88 | KP278946 | Schlautman et al. (2015) | GTAGGTATCATCGCTGTCTTC | GTGCATCACATACAAGCTCT |
| SCF64185 | 11 | 36.88 | KP278745 | Schlautman et al. (2015) | CACCTCATTTGGTTCATTCT | CAGATACTAAAGGTTGCCGTA |
| SCF183590 | 11 | 37.5 | KP278933 | Schlautman et al. (2015) | TTTGTAGTATGGGGACACTGAT | AAAGAGGCAGGTCAGAAAAT |
| SCF21596 | 11 | 39.89 | KP278655 | Schlautman et al. (2015) | ATATACTGGCATAAACACCCTC | CCTTACTCTTATCATGGCTAGG |
| vm68798 | 11 | 47.62 | JF834242 | Zhu et al. (2012) | ATAGAAATCGAGCAAGGAGAG | CAGACCTAAACTCAATTTCTGG |
| SCF132506 | 11 | 50.06 | KP278862 | Schlautman et al. (2015) | AATGTGCCAAGTTTTGTAGAC | GTCCCCTATAAGTCATCTGAAA |
| SCF57497 | 11 | 50.63 | KP278737 | Schlautman et al. (2015) | ATCTGTAGGTTGTGTTACCCC | ATCAACTGTATCTACCCACCAA |
| SCF120937 | 11 | 53 | KP278844 | Schlautman et al. (2015) | TGTGCAAGAGTCATCTCCTAT | TATTCCCTTTCATTCTCCTTC |
| 482_K70 | 11 | 53.59 | KP279193 | Schlautman et al. (2015) | ACAGCGGCATAGTAAAATGA | GTCACCGAAATCTCACTCAATA |
| SCF123189 | 11 | 53.59 | KP278847 | Schlautman et al. (2015) | CCTAGAAATGTTACTCTCCGAC | TTCACTTCCTTACTCCTTTCAT |
| SCF136826 | 11 | 53.59 | KP278868 | Schlautman et al. (2015) | GATCTTGATTAGCTCCAACTG | GCTTACACCAATTCTACAGTCA |
| SCF158255 | 11 | 53.59 | KP278905 | Schlautman et al. (2015) | ATGCGTACACCTCAATCTTT | GTGGGTACTTGTTTTCAGTTC |
| SCF66313 | 11 | 53.59 | KP279032 | Schlautman et al. (2015) | AATTTGACCCTCTTTCCCT | GTCCAAAATACACAAACTAGCC |
| SCF95754 | 11 | 53.59 | KP278797 | Schlautman et al. (2015) | CAGTGAGACTTCAGCTTGATAC | ATTGGTGACTTAGGAGTGAGAC |
| 1trimcontig444344 | 11 | 54.19 | KP279252 | Schlautman et al. (2015) | CTGCTAATGTTGTTTGTTGC | TATTATCTCCCACCTAATGAGC |
| 308539_K70 | 11 | 54.19 | KP279209 | Schlautman et al. (2015) | CTAAATTCTCAACATCTCTGGC | CCAAGAAGCATAAGGGATAGT |
| SCF161998 | 11 | 57.76 | KP278911 | Schlautman et al. (2015) | ATATACCAGTGCTCTTTCCATC | AGACTTCTTTCTCCAAAGGC |
| SCF150173 | 11 | 64.54 | KP278894 | Schlautman et al. (2015) | GTGTTGGGAAACAGCAGAT | TTATTCTCGTTGTCAGCCTT |
| SCF110757 | 11 | 66.26 | KP278825 | Schlautman et al. (2015) | TCATATCAACCTAACAATCGG | CACAAACAAGGAAATTAAGACC |
| SCF1047 | 11 | 72.22 | KP278596 | Schlautman et al. (2015) | GAGCTTTGGCCTCATATTACT | CGAATTACTCCAACCAACAT |
| SCF28931 | 11 | 73.75 | KP278678 | Schlautman et al. (2015) | TCTCATAAGTCAGAACCTCACA | CTAAACTAAACCTCCTAACCGA |
| SCF160647 | 11 | 77.45 | KP278909 | Schlautman et al. (2015) | TAACTCAAAGAACCTAACCCC | TAAAGTGACAGGTAATGTCGTC |
| SCF181772 | 11 | 81.02 | KP278931 | Schlautman et al. (2015) | AGCAACGTATGGTGGTATC | CATTTGTTTCCACAGCTTC |
| SCF118608 | 11 | 84.31 | KP278841 | Schlautman et al. (2015) | AACTACTCGATCTTCACCCTTA | AGGAGACCAACACTTAACCTC |
| SCF28509 | 11 | 93.27 | KP278676 | Schlautman et al. (2015) | GCAAACACCACACTATATGAGA | ATAGAGAACCACAGAACAGGAC |
| 1trimcontig175770 | 12 | 2.84 | KP279220 | Schlautman et al. (2015) | GTGTAGCTTGGAAAATAGGAGT | ACTAGGGAGCGAGAGAGAGTA |
| 411145_K63 | 12 | 2.84 | KP279179 | Schlautman et al. (2015) | GGTAGGAATTAAAGTGAAGACG | ACTAGGGAGCGAGAGAGAGTA |
| ct140233 | 12 | 2.84 | KP279126 | Schlautman et al. (2015) | TTACAGAAGGAAGAGAGAGGAA | ACTGGCTTCTATAGCTCATTTC |
| Ig28559a | 12 | 2.84 |  | Georgi et al. (2013) | CAAGAGTCGCAAATTCCACA | CCTCCTTCTAGAGAGGGCCA |
| SCF28279 | 12 | 2.84 | KP278675 | Schlautman et al. (2015) | GATACTTTACCTCCTCCTCAAG | TTGTCCTCTATCTCTAACTCCC |
| 417587_K63 | 12 | 5.82 | KP279187 | Schlautman et al. (2015) | TGGGTAGATATTAGATGGCAGT | CTTCTTCTGGAAATCTGGTTAG |
| SCF28955 | 12 | 5.82 | KP278679 | Schlautman et al. (2015) | TATTCAAAGCCACTAGGCAC | CAAACCAAATTCTCCTTCTG |
| ct174735 | 12 | 6.26 | KP279147 | Schlautman et al. (2015) | CTTATTTGTATGGCCTTCCT | GCAGCATATATTGTCCAGTTC |
| SCF160663 | 12 | 6.85 | KP278910 | Herein | TTACACCCTATCTCCTGTTTTC | CAGTTCATCTTGCTAGTTATGC |
| ct110752 | 12 | 8.25 | KP279110 | Schlautman et al. (2015) | ACACACACTAACGAAATCCTTC | CTAGCTCCGACATTGTTATCTC |
| SCF116485 | 12 | 8.25 | KP278834 | Schlautman et al. (2015) | CAATATAAACGTCAGTCACCAG | ACTTTTGGTTATGCTGGAAG |
| scf6355 | 12 | 8.25 |  | Georgi et al. (2013) | ACAATGTTGTCATTCCGACG | CTAGACTCGTCCAAAAGCCC |
| scaffold_111040 | 12 | 9.82 |  | Herein | ACACAGTTTATGCAGAGCTTAC | ATCTCTCTCTCTCTCTTTGAGC |
| scf2882 | 12 | 9.82 |  | Georgi et al. (2013) | CGCTACCATTGTCAGCTTCA | ACACTCAAAAGCAGGTGGCT |
| scf4b | 12 | 14.77 |  | Georgi et al. (2013) | GATACGATACGGATACGCGG | GTCGATCATGGTCGTCAGTG |
| scaffold_42843 | 12 | 17.15 |  | Herein | TTTCTCCATCTCTCTCTTATCC | ACTCACTGTTCACCTTCTCTG |
| scaffold01187 | 12 | 17.15 |  | Blueberry Flowering Gene | AATGTTTTGTGTCTCCCAATCC | CATTATTTAGCACCAGCGTTGT |
| SCF122440 | 12 | 24.86 | KP279066 | Schlautman et al. (2015) | CTAATCTTCCTCCTCTTGTTGA | CGACAAACTAACATATCATCTCC |
| SCF34071 | 12 | 35.42 | KP278694 | Schlautman et al. (2015) | CGTGTGCAGATTTACTTCAG | AATTCATAGATCCCCATGAC |
| SCF59739 | 12 | 38.3 | KP278741 | Schlautman et al. (2015) | GTATGACTGTACCAAACAAACC | CAGCTTTCCCTTCTAAATGA |
| SCF190609 | 12 | 43.72 |  | Herein | TAACGGAAAGAGTTAAAGGAC | TTGTATCAGAGAAGAAGGAGG |
| SCF46588 | 12 | 43.72 | KP278714 | Schlautman et al. (2015) | ACAAACCTTGAGCCTATTTG | GTCTGAGTTTCCACTATCGTCT |
| SCF98180 | 12 | 43.72 | KP278802 | Schlautman et al. (2015) | CTCCTCTGCTTATCTCTTCAAC | GGTTTTCCCTTCTCAAGATTAC |
| SCF13753 | 12 | 47.95 | KP278641 | Schlautman et al. (2015) | AAGTCCTTTCCTTCTTTTGC | GCTATGTGATGTCGTTCCTAA |
| SCF14189 | 12 | 47.95 | KP278644 | Schlautman et al. (2015) | GTCTAGGTGAGGATGGTTGAT | AAAACAGAGCCCAACAAGT |
| SCF6926 | 12 | 47.95 | KP278615 | Schlautman et al. (2015) | ACATGCACTTCAAATAGTACCC | TTACAACTTACACAGGAAGCAG |
| SCF76310 | 12 | 47.95 | KP278762 | Schlautman et al. (2015) | CTGTGTAGAACTGCATCAAAAC | TCCTAGAGACCAACCCAATAC |
| SCF96306 | 12 | 49.26 | KP279050 | Schlautman et al. (2015) | CCTGTAGTGAGTTACCTTCCAT | GCTGTCAACCATCCATTATT |
| SCF37628 | 12 | 49.43 | KP278700 | Schlautman et al. (2015) | ACCAGCTCAGATAACAATGC | GAGTAGGATACCTCCACACCTA |
| scf37h | 12 | 51.8 | JN230518 | Georgi et al. (2013) | TGGACTTTTCTTGCTTGGCT | GGATACACGTGACCGAGCTT |
| 309084_K70 | 12 | 52.87 | KP279212 | Schlautman et al. (2015) | CTTCTTTTCCTCTCCACTGATA | CTCTCCGTTGTCCATTTCT |
| SCF34584 | 12 | 52.87 | KP278696 | Schlautman et al. (2015) | GTCTGTTTGGAAGAAGAAGGT | CTGTTCGTCAATCCCTAGC |
| vm26877 | 12 | 55.31 | JF834264 | Zhu et al. (2012) | CCCCTTTTGAACGAAACTATAC | ACATCTCAATTCCGAGCATA |
| ct129169 | 12 | 56.43 | KP279119 | Schlautman et al. (2015) | TAAATCACCTTCTTCCTCCTC | GGTCCCAAACTTACTACTCAAA |
| SCF16166 | 12 | 56.43 | KP278646 | Schlautman et al. (2015) | CCTAGTCATTCTTCTACTCCCA | GGGTTATCTCGTCCATATTGT |
| 354699_K63 | 12 | 59.41 | KP279172 | Schlautman et al. (2015) | GAAGCGATTTGGAAGAAAC | ACACAGAGAGATTACGAACACA |
| SCF69698 | 12 | 59.41 | KP278751 | Schlautman et al. (2015) | GAGGAGATAAAGGTTTGTTGAG | CTTTGAGACTTTGAGTGAGACA |
| SCF11065 | 12 | 61.26 | KP278634 | Schlautman et al. (2015) | CTTTGTCCCAACACGTTAAT | AAGTCTATAAGCATCCTGCAAC |
| SCF149989 | 12 | 61.26 | KP278892 | Schlautman et al. (2015) | AGTAGGCATTGTTCACTCACTC | TTTCTCCTAAAGCTAAACTCCC |
| SCF38430 | 12 | 61.26 | KP278702 | Schlautman et al. (2015) | CAATAGTTAGGAAGTTGGAACC | CTAAGAACCAAACAGAGCCTTA |
| 76126_K63 | 12 | 61.79 | KP279162 | Schlautman et al. (2015) | TTTATTGGAGCGAAAGAGAG | AAAAGGGGAGGAGAGAGAT |
| SCF138394 | 12 | 61.79 | KP278871 | Schlautman et al. (2015) | AAGCCCAGAAGAAATAACCTA | TGCAAATGTTAGGAACTGTGT |
| 418596_K63 | 12 | 62.39 | KP279190 | Schlautman et al. (2015) | CGTGAGTTTGAGTGAGTAATTG | AGGACATGGTGAGTTGAGAAT |
| scf15b | 12 | 62.39 |  | Georgi et al. (2013) | CTGCCTTGTTTCCCTCTCTG | GGATTGGTTTGTTGGTCGTC |
| SCF38942 | 12 | 62.39 | KP278704 | Schlautman et al. (2015) | CTTGCTATTTGGTACTCGTCTT | CTTGACAGTTATTTCTCTTCGG |
| SCF116567 | 12 | 64.09 | KP278835 | Schlautman et al. (2015) | GTTGGTCTACAATTCTGTTCCT | GCCCTTTTAGTTGAAATGC |
| 408825_K63 | 12 | 65.37 | KP279176 | Schlautman et al. (2015) | GTTCTCCTCTTTCATCATTCAG | AGTCTTGAACTCCTTGTACTCG |
| ct144936 | 12 | 65.37 | KP279129 | Schlautman et al. (2015) | AGGTGACTAAGGCAGTGTTC | CGTGTCTGTTTGGTTAGTAGGT |
| vm40600 | 12 | 65.37 | JF834274 | Zhu et al. (2012) | CAAAAGAGCCATGAAATAGG | TTGGTGAAAACTATACCTGTCC |
| scf79c | 12 | 66.36 |  | Georgi et al. (2013) | GGTTCTTCGTGGCATGATAGT | CCAAATAACCCAGGAGAGCA |
| SCF117422 | 12 | 72.34 | KP278839 | Schlautman et al. (2015) | TTCTGTTTCTTGGCTCTGTATC | TATTATGCTACATCGGTCGAG |
| SCF159195 | 12 | 72.34 | KP278908 | Schlautman et al. (2015) | AACAAAGACCCTAATCAGACAC | ACAATCAAAACACCGTCAG |
| scf6213 | 12 | 77.11 |  | Georgi et al. (2013) | GCTCGCTCTCGCATATTTTC | CCTAGCCCGTTCATCATTGT |
| SCF75572 | 12 | 77.11 | KP278761 | Schlautman et al. (2015) | GACAAGTGGTTGGGGATAC | ACCCTCATCATCACTCCTT |
| vm34671 | 12 | 79.7 | JF834271 | Zhu et al. (2012) | CCATCTCTCTCTTGTTTCTCTC | GCGAAAAATAAGTCTCCACA |
| SCF101914 | 12 | 85.12 | KP278806 | Schlautman et al. (2015) | CTTTGGAGCACAACAACTCTA | GTGTAAAGACCAGGACCCTT |
| SCF145689 | 12 | 85.12 | KP278885 | Schlautman et al. (2015) | GGCATAAGAGTAGACCATGAAC | GTACTATAAAATGCTTCCAGCG |
| SCF169090 | 12 | 85.12 | KP278917 | Schlautman et al. (2015) | GAGACAAAGTTCAAATAGGGAG | ATACTGCAACCGATACTGAGA |
| scf248 | 12 | 85.12 |  | Georgi et al. (2013) | CAACTGGAGGCAAAACAACA | CACGCATTGCAATTATACCG |
| SCF102190 | 12 | 86.61 | KP279054 | Schlautman et al. (2015) | GAGGAAAGGGTGAGAGTTTT | GTTTGACGAAAAGGAGACTG |
| SCF3191 | 12 | 86.61 | KP278603 | Schlautman et al. (2015) | GCACTATCAGGAAGAGGAATTA | GTAACACCAGAAAACAACTGC |
| SCF3261 | 12 | 86.61 | KP278604 | Schlautman et al. (2015) | GTTTACCATATTCACTCCTTCC | TGAGACAGACCTAACATTTGAC |
| scaffold_84992 | 12 | 90.6 |  | Herein | TATACAGTTTGCTCGTTGGAC | CGATCCACTAACACAAAAGAAC |
