# Supplementary material for: Construction of a High-Density American Cranberry (Vaccinium macrocarpon Ait.) Composite Map Using Genotyping-by-Sequencing for Multi-pedigree Linkage Mapping
Source: G3 (Bethesda). 2017 Mar 1;7(4):1177–89. doi: 10.1534/g3.116.037556 (PMC5386866; doi:10.1534/g3.116.037556)
Supplement: Supplementary file 5 [file 1177TableS2.docx]

Table S2. Features of the parental bin maps and linkage groups (LGs) constructed for the maternal parent (M), [BGx(BLxNL)]95, and the paternal parent (P), GH1x35, for the GRYG full-sib mapping population using simple sequence repeats (SSRs) and single nucleotide polymorphisms (SNPs).

| LG | Length (cM) | | SNPs | | SSRs | | Markers^a^ | | Bins^b^ | | Bins with SSRs^c^ | | Mean Gap^d^ | | Mean Recombination^e^ | |
| --- | --- | --- | --- | --- | --- | --- | --- | --- | --- | --- | --- | --- | --- | --- | --- | --- |
|  | M | P | M | P | M | P | M | P | M | P | M | P | M | P | M | P |
| LG1 | 126.1 | 111.0 | 227 | 254 | 5 | 6 | 232 | 260 | 58 | 64 | 5 | 5 | 2.2 | 1.8 | 1.3 | 1.1 |
| LG2 | 95.0 | 90.6 | 160 | 125 | 4 | 1 | 164 | 126 | 40 | 32 | 4 | 1 | 2.4 | 2.9 | 0.9 | 0.9 |
| LG3 | 102.8 | 86.4 | 130 | 139 | 5 | 10 | 135 | 149 | 37 | 28 | 5 | 8 | 2.9 | 3.2 | 1.0 | 0.9 |
| LG4 | 90.9 | 85.2 | 132 | 150 | 11 | 12 | 143 | 162 | 33 | 44 | 10 | 11 | 2.8 | 2.0 | 0.9 | 0.9 |
| LG5 | 61.8 | 88.0 | 30 | 162 | 4 | 13 | 34 | 175 | 10 | 37 | 4 | 10 | 6.9 | 2.4 | 0.6 | 0.9 |
| LG6 | 110.7 | 84.2 | 173 | 184 | 8 | 5 | 181 | 189 | 46 | 43 | 7 | 3 | 2.5 | 2.0 | 1.1 | 0.8 |
| LG7 | 106.1 | 85.4 | 135 | 132 | 10 | 6 | 145 | 138 | 40 | 37 | 8 | 4 | 2.7 | 2.4 | 1.1 | 0.8 |
| LG8 | 73.7 | 79.5 | 95 | 106 | 6 | 3 | 101 | 109 | 23 | 36 | 4 | 3 | 3.4 | 2.3 | 0.7 | 0.8 |
| LG9 | 97.6 | 89.9 | 197 | 194 | 6 | 3 | 203 | 197 | 40 | 45 | 6 | 3 | 2.5 | 2.0 | 1.0 | 0.9 |
| LG10 | 93.5 | 77.3 | 128 | 159 | 5 | 3 | 133 | 162 | 39 | 28 | 5 | 1 | 2.5 | 2.9 | 0.9 | 0.8 |
| LG11 | 111.0 | 94.1 | 162 | 213 | 7 | 6 | 169 | 219 | 42 | 43 | 6 | 6 | 2.7 | 2.2 | 1.1 | 0.9 |
| LG12 | 99.4 | 88.8 | 180 | 198 | 11 | 13 | 191 | 211 | 41 | 35 | 7 | 10 | 2.5 | 2.6 | 1.0 | 0.9 |
| **Mean** | **97.4** | **88.4** | **146** | **168** | **7** | **7** | **153** | **175** | **37** | **39** | **6** | **5** | **3.0** | **2.4** | **1.0** | **0.9** |
| **Total** | **1168.6** | **1060.4** | **1749** | **2016** | **82** | **81** | **1831** | **2097** | **449** | **472** | **71** | **65** |  |  |  |  |

^a^ Total number of SNPs and SSRs mapped

^b^ Total number of unique marker bins estimated using the ASMap package in R (Taylor and Butler 2015).

^c^ Number of unique marker bins that contained at least one SSR

^d^ Mean distance between unique marker bins

^e^ Mean number of recombination events per progeny per parental LG
