# Supplementary material for: Construction of a High-Density American Cranberry (Vaccinium macrocarpon Ait.) Composite Map Using Genotyping-by-Sequencing for Multi-pedigree Linkage Mapping
Source: G3 (Bethesda). 2017 Mar 1;7(4):1177–89. doi: 10.1534/g3.116.037556 (PMC5386866; doi:10.1534/g3.116.037556)
Supplement: Supplementary file 6 [file 1177TableS3.docx]

Table S3. Features of the parental bin maps and linkage groups (LGs) constructed for the maternal parent (M), Mullica Queen, and the paternal parent (P), Crimson Queen, for the CNJ02 full-sib mapping population using simple sequence repeats (SSRs) and single nucleotide polymorphisms (SNPs).

| LG | Length (cM) | | SNPs | | SSRs | | Markers^a^ | | Bins^b^ | | Bins with SSRs^c^ | | Mean Gap^d^ | | Mean Recombination^e^ | |
| --- | --- | --- | --- | --- | --- | --- | --- | --- | --- | --- | --- | --- | --- | --- | --- | --- |
|  | M | P | M | P | M | P | M | P | M | P | M | P | M | P | M | P |
| LG1 | 107.0 | 79.3 | 202 | 198 | 31 | 35 | 233 | 233 | 44 | 43 | 18 | 17 | 2.5 | 1.9 | 1.1 | 0.8 |
| LG2 | 113.8 | 100.2 | 161 | 156 | 45 | 45 | 206 | 201 | 51 | 49 | 26 | 30 | 2.3 | 2.1 | 1.1 | 1.0 |
| LG3 | 111.8 | 87.8 | 152 | 161 | 41 | 39 | 193 | 200 | 47 | 42 | 23 | 22 | 2.4 | 2.1 | 1.1 | 0.9 |
| LG4 | 115.7 | 81.0 | 150 | 138 | 54 | 49 | 204 | 187 | 56 | 38 | 31 | 25 | 2.1 | 2.2 | 1.2 | 0.8 |
| LG5 | 105.0 | 88.9 | 141 | 179 | 27 | 29 | 168 | 208 | 40 | 41 | 19 | 16 | 2.7 | 2.2 | 1.1 | 0.9 |
| LG6 | 111.4 | 82.5 | 172 | 177 | 38 | 36 | 210 | 213 | 45 | 35 | 24 | 23 | 2.5 | 2.4 | 1.1 | 0.8 |
| LG7 | 108.2 | 87.6 | 182 | 165 | 46 | 45 | 228 | 210 | 51 | 46 | 31 | 29 | 2.2 | 2.0 | 1.1 | 0.9 |
| LG8 | 112.1 | 74.7 | 133 | 147 | 40 | 41 | 173 | 188 | 48 | 39 | 23 | 24 | 2.4 | 2.0 | 1.1 | 0.7 |
| LG9 | 107.9 | 73.3 | 200 | 185 | 42 | 41 | 242 | 226 | 45 | 45 | 19 | 21 | 2.5 | 1.7 | 1.1 | 0.7 |
| LG10 | 91.4 | 76.3 | 140 | 144 | 39 | 38 | 179 | 182 | 39 | 38 | 23 | 19 | 2.4 | 2.1 | 0.9 | 0.8 |
| LG11 | 105.7 | 87.2 | 206 | 193 | 31 | 31 | 237 | 224 | 45 | 39 | 23 | 17 | 2.4 | 2.3 | 1.1 | 0.9 |
| LG12 | 106.5 | 60.9 | 163 | 177 | 48 | 38 | 211 | 215 | 40 | 31 | 26 | 20 | 2.7 | 2.0 | 1.1 | 0.6 |
| **Mean** | **108.0** | **81.6** | **167** | **168** | **40** | **39** | **207** | **207** | **46** | **41** | **24** | **22** | **2.4** | **2.1** | **1.1** | **0.8** |
| **Total** | **1296.5** | **979.7** | **2002** | **2020** | **482** | **467** | **2484** | **2487** | **551** | **486** | **286** | **263** |  |  |  |  |

^a^ Total number of SNPs and SSRs mapped

^b^ Total number of unique marker bins estimated using the ASMap package in R (Taylor and Butler 2015).

^c^ Number of unique marker bins that contained at least one SSR

^d^ Mean distance between unique marker bins

^e^ Mean number of recombination events per progeny per parental LG
