# Supplementary material for: Construction of a High-Density American Cranberry (Vaccinium macrocarpon Ait.) Composite Map Using Genotyping-by-Sequencing for Multi-pedigree Linkage Mapping
Source: G3 (Bethesda). 2017 Mar 1;7(4):1177–89. doi: 10.1534/g3.116.037556 (PMC5386866; doi:10.1534/g3.116.037556)
Supplement: Supplementary file 7 [file 1177TableS4.docx]

Table S4. Features of the parental bin maps and linkage groups (LGs) constructed for the maternal parent (M), Mullica Queen, and the paternal parent (P), Stevens, for the CNJ04 full-sib mapping population using simple sequence repeats (SSRs) and single nucleotide polymorphisms (SNPs).

| LG | Length (cM) | | SNPs | | SSRs | | Markers^a^ | | Bins^b^ | | Bins with SSRs^c^ | | Mean Gap^d^ | | Mean Recombination^e^ | |
| --- | --- | --- | --- | --- | --- | --- | --- | --- | --- | --- | --- | --- | --- | --- | --- | --- |
|  | M | P | M | P | M | P | M | P | M | P | M | P | M | P | M | P |
| LG1 | 117.1 | 89.9 | 171 | 197 | 6 | 9 | 177 | 206 | 28 | 32 | 6 | 6 | 4.3 | 2.9 | 1.2 | 0.9 |
| LG2 | 103.4 | 78.3 | 158 | 122 | 16 | 16 | 174 | 138 | 31 | 27 | 10 | 12 | 3.5 | 3.0 | 1.0 | 0.8 |
| LG3 | 61.7 | 59.9 | 114 | 138 | 8 | 9 | 122 | 147 | 16 | 19 | 5 | 7 | 4.1 | 3.3 | 0.6 | 0.6 |
| LG4 | 83.0 | 55.6 | 114 | 152 | 8 | 11 | 122 | 163 | 20 | 17 | 7 | 10 | 4.4 | 3.5 | 0.8 | 0.6 |
| LG5 | 88.6 | 85.1 | 106 | 145 | 7 | 3 | 113 | 148 | 23 | 21 | 5 | 3 | 4.0 | 4.3 | 0.9 | 0.8 |
| LG6 | 98.9 | 77.8 | 161 | 166 | 10 | 15 | 171 | 181 | 31 | 25 | 9 | 13 | 3.3 | 3.2 | 1.0 | 0.8 |
| LG7 | 108.0 | 72.1 | 128 | 111 | 8 | 6 | 136 | 117 | 22 | 22 | 7 | 4 | 5.1 | 3.4 | 1.1 | 0.7 |
| LG8 | 88.6 | 56.9 | 115 | 101 | 8 | 11 | 123 | 112 | 21 | 21 | 6 | 9 | 4.4 | 2.8 | 0.9 | 0.6 |
| LG9 | 97.5 | 69.5 | 146 | 141 | 13 | 12 | 159 | 153 | 21 | 17 | 11 | 9 | 4.9 | 4.3 | 1.0 | 0.7 |
| LG10 | 70.9 | 75.1 | 101 | 139 | 14 | 15 | 115 | 154 | 22 | 23 | 10 | 9 | 3.4 | 3.4 | 0.7 | 0.8 |
| LG11 | 99.5 | 55.8 | 188 | 95 | 8 | 9 | 196 | 104 | 29 | 13 | 7 | 5 | 3.6 | 4.7 | 1.0 | 0.6 |
| LG12 | 69.2 | 69.2 | 152 | 168 | 14 | 15 | 166 | 183 | 19 | 21 | 10 | 11 | 3.9 | 3.5 | 0.7 | 0.7 |
| **Mean** | **90.5** | **70.4** | **138** | **140** | **10** | **11** | **148** | **151** | **24** | **22** | **8** | **8** | **4.1** | **3.5** | **0.9** | **0.7** |
| **Total** | **1086.4** | **845.2** | **1654** | **1675** | **120** | **131** | **1774** | **1806** | **283** | **258** | **93** | **98** |  |  |  |  |

^a^ Total number of SNPs and SSRs mapped

^b^ Total number of unique marker bins estimated using the ASMap package in R (Taylor and Butler 2015).

^c^ Number of unique marker bins that contained at least one SSR

^d^ Mean distance between unique marker bins

^e^ Mean number of recombination events per progeny per parental LG
