# Supplementary material for: Construction of a High-Density American Cranberry (Vaccinium macrocarpon Ait.) Composite Map Using Genotyping-by-Sequencing for Multi-pedigree Linkage Mapping
Source: G3 (Bethesda). 2017 Mar 1;7(4):1177–89. doi: 10.1534/g3.116.037556 (PMC5386866; doi:10.1534/g3.116.037556)
Supplement: Supplementary file 8 [file 1177TableS5.docx]

Table S5. Total number of markers, by linkage group (LG), mapped in the parental component bin maps for each of the three cranberry full-sib populations (i.e. GRYG, CNJ02, and CNJ04).

| LG | # SNPs | | | # SSRs | | | # Total Markers | | |
| --- | --- | --- | --- | --- | --- | --- | --- | --- | --- |
|  | GRYG | CNJ02 | CNJ04 | GRYG | CNJ02 | CNJ04 | GRYG | CNJ02 | CNJ04 |
| LG1 | 391 | 327 | 286 | 10 | 43 | 10 | 401 | 370 | 296 |
| LG2 | 243 | 261 | 240 | 5 | 57 | 21 | 248 | 318 | 261 |
| LG3 | 231 | 265 | 213 | 12 | 51 | 12 | 243 | 316 | 225 |
| LG4 | 239 | 236 | 243 | 17 | 59 | 13 | 256 | 295 | 256 |
| LG5 | 181 | 257 | 235 | 13 | 34 | 7 | 194 | 291 | 242 |
| LG6 | 291 | 296 | 281 | 9 | 47 | 16 | 300 | 343 | 297 |
| LG7 | 223 | 289 | 211 | 15 | 58 | 8 | 238 | 347 | 219 |
| LG8 | 169 | 226 | 198 | 9 | 52 | 12 | 178 | 278 | 210 |
| LG9 | 308 | 323 | 262 | 8 | 52 | 14 | 316 | 375 | 276 |
| LG10 | 241 | 232 | 219 | 6 | 47 | 19 | 247 | 279 | 238 |
| LG11 | 322 | 323 | 246 | 10 | 41 | 11 | 332 | 364 | 257 |
| LG12 | 319 | 291 | 280 | 17 | 58 | 24 | 336 | 349 | 304 |
| **Mean** | **263** | **277** | **243** | **11** | **50** | **14** | **274** | **327** | **257** |
| **Total** | **3158** | **3326** | **2914** | **131** | **599** | **167** | **3289** | **3925** | **3081** |
