# Supplementary material for: Construction of a High-Density American Cranberry (Vaccinium macrocarpon Ait.) Composite Map Using Genotyping-by-Sequencing for Multi-pedigree Linkage Mapping
Source: G3 (Bethesda). 2017 Mar 1;7(4):1177–89. doi: 10.1534/g3.116.037556 (PMC5386866; doi:10.1534/g3.116.037556)
Supplement: Supplementary file 9 [file 1177TableS6.docx]

Table S6. Statistic generate during composite map construction with the six parental component bin maps from the cranberry GRYG, CNJ02, and CNJ04 populations using LPmerge (Endelman and Plomion 2014). The max interval size, *k*, which minimized the root mean square error (RMSE) is displayed along with the corresponding RMSE and standard deviation (SD).

| LG | Max Interval (*k*) | Number of Loci | Map Length | RMSE | SD |
| --- | --- | --- | --- | --- | --- |
| 1 | 5 | 638 | 115.88 | 10.8 | 7.99 |
| 2 | 9 | 479 | 100.75 | 6.68 | 3.37 |
| 3 | 4 | 461 | 92.4 | 8.66 | 4.94 |
| 4 | 4 | 484 | 86.35 | 8.53 | 7.99 |
| 5 | 9 | 440 | 93.09 | 6.45 | 3.68 |
| 6 | 10 | 523 | 93.77 | 7.58 | 3.27 |
| 7 | 10 | 477 | 97.03 | 7.79 | 3.99 |
| 8 | 10 | 424 | 85.2 | 10.06 | 4.64 |
| 9 | 4 | 586 | 89.86 | 9.14 | 6.54 |
| 10 | 6 | 429 | 84.11 | 3.25 | 1.35 |
| 11 | 10 | 541 | 95.25 | 6.17 | 3.57 |
| 12 | 5 | 591 | 90.6 | 9.27 | 3.49 |
