# Supplementary material for: Construction of a High-Density American Cranberry (Vaccinium macrocarpon Ait.) Composite Map Using Genotyping-by-Sequencing for Multi-pedigree Linkage Mapping
Source: G3 (Bethesda). 2017 Mar 1;7(4):1177–89. doi: 10.1534/g3.116.037556 (PMC5386866; doi:10.1534/g3.116.037556)
Supplement: Supplementary file 10 [file 1177TableS7.docx]

Table S7. The number of gaps between unique marker positions in the cranberry consensus map exceeding 1, 2, 3, 4, and 5 cM in length by linkage group (LG).

| LG | Gap Size | | | | |
| --- | --- | --- | --- | --- | --- |
|  | 1 cM | 2 cM | 3 cM | 4 cM | 5 cM |
| LG1 | 40 | 9 | 2 | 0 | 0 |
| LG2 | 28 | 12 | 3 | 1 | 1 |
| LG3 | 35 | 13 | 4 | 0 | 0 |
| LG4 | 28 | 6 | 3 | 2 | 1 |
| LG5 | 31 | 10 | 5 | 1 | 0 |
| LG6 | 31 | 7 | 2 | 1 | 0 |
| LG7 | 29 | 7 | 1 | 0 | 0 |
| LG8 | 28 | 8 | 2 | 0 | 0 |
| LG9 | 31 | 9 | 2 | 1 | 0 |
| LG10 | 27 | 7 | 3 | 2 | 1 |
| LG11 | 37 | 5 | 0 | 0 | 0 |
| LG12 | 40 | 11 | 2 | 1 | 0 |
| **mean** | **32.1** | **8.7** | **2.4** | **0.8** | **0.3** |
| **total** | **385** | **104** | **29** | **9** | **3** |
