# Supplementary material for: Construction of a High-Density American Cranberry (Vaccinium macrocarpon Ait.) Composite Map Using Genotyping-by-Sequencing for Multi-pedigree Linkage Mapping
Source: G3 (Bethesda). 2017 Mar 1;7(4):1177–89. doi: 10.1534/g3.116.037556 (PMC5386866; doi:10.1534/g3.116.037556)
Supplement: Supplementary file 11 [file 1177TableS8.docx]

Table S8. Total number of cranberry scaffold, by linkage group (LG), from the Polashock *et al.* (2014) assembly containing SNPs or SSRs that were anchored in the cranberry composite map. The number of anchored scaffolds containing predicted coding DNA sequences (CDS) and total number of base pairs (bp) contained within those scaffolds is also provided.

| LG | Anchored scaffolds | Length of anchored scaffolds (bp) | Anchored scaffolds containing CDS |
| --- | --- | --- | --- |
| LG1 | 431 | 2407463 | 186 |
| LG2 | 325 | 1840095 | 147 |
| LG3 | 299 | 1535779 | 118 |
| LG4 | 326 | 1815950 | 140 |
| LG5 | 289 | 1542819 | 125 |
| LG6 | 345 | 2014976 | 155 |
| LG7 | 305 | 1672098 | 129 |
| LG8 | 284 | 1576566 | 112 |
| LG9 | 384 | 2032876 | 149 |
| LG10 | 281 | 1517934 | 110 |
| LG11 | 349 | 1845278 | 126 |
| LG12 | 365 | 2006173 | 157 |
| **mean** | **332** | **1817334** | **138** |
| **total** | **3983** | **21808007** | **1654** |
