# Supplementary material for: Construction of a High-Density American Cranberry (Vaccinium macrocarpon Ait.) Composite Map Using Genotyping-by-Sequencing for Multi-pedigree Linkage Mapping
Source: G3 (Bethesda). 2017 Mar 1;7(4):1177–89. doi: 10.1534/g3.116.037556 (PMC5386866; doi:10.1534/g3.116.037556)
Supplement: Supplementary file 12 [file 1177TableS9.docx]

Table S9. Pair-wise Spearman rank correlations between the linkage groups (LGs) the cranberry composite map and previous cranberry linkage maps (Schlautman *et al.* 2015; Covarrubias-Pazaran *et al.* 2016).

| LG | Composite vs. Schlautman *et al.* (2015a) | Composite vs. Covarrubias-Pazaran *et al.* (2016) |
| --- | --- | --- |
| LG1 | 0.99 | 0.99 |
| LG2 | 1 | 1 |
| LG3 | 1 | 0.96 |
| LG4 | 0.99 | 1 |
| LG5 | 1 | 1 |
| LG6 | 1 | 0.99 |
| LG7 | 0.99 | 0.99 |
| LG8 | 1 | 0.97 |
| LG9 | 1 | 0.93 |
| LG10 | 1 | 0.98 |
| LG11 | 0.99 | 0.94 |
| LG12 | 0.99 | 0.96 |
| **mean** | **0.996** | **0.976** |
