# Supplementary material for: Construction of a High-Density American Cranberry (Vaccinium macrocarpon Ait.) Composite Map Using Genotyping-by-Sequencing for Multi-pedigree Linkage Mapping
Source: G3 (Bethesda). 2017 Mar 1;7(4):1177–89. doi: 10.1534/g3.116.037556 (PMC5386866; doi:10.1534/g3.116.037556)
Supplement: Supplementary file 13 [file 1177TableS10.docx]

Table S10. Proportion of markers in the linkage groups (LGs) of the 6 parental component maps (i.e. [BGx(BLxNL)]95, GH1x35, Mullica Queen (MQ), Crimson Queen (CQ), and Stevens (ST) from the GRYG, CNJ02, and CNJ04 populations) that display significant segregation distortion from the expected Mendelian genotype rations according to χ2 tests at the *p* < 0.1 level.

| LG | [BGx(BLxNL)]95 | GH1x35 | MQ-CNJ02 | CQ | MQ-CNJ04 | ST |
| --- | --- | --- | --- | --- | --- | --- |
| LG1 | 0 | 0.21 | 0.1 | 0 | 0.06 | 0 |
| LG2 | 0 | 0.01 | 0.21 | 0 | 0 | 0.17 |
| LG3 | 0.06 | 0 | 0.05 | 0 | 0 | 0 |
| LG4 | 0 | 0 | 0 | 0 | 0.13 | 0 |
| LG5 | 0.06 | 0 | 0.01 | 0.07 | 0 | 0 |
| LG6 | 0 | 0.81 | 0.13 | 0.06 | 0 | 0 |
| LG7 | 0.13 | 0.72 | 0 | 0 | 0 | 0.03 |
| LG8 | 0.14 | 0 | 0 | 0.37 | 0 | 0 |
| LG9 | 0.13 | 0.37 | 0 | 0 | 0 | 0.1 |
| LG10 | 0.02 | 0.3 | 0.35 | 0 | 0 | 0 |
| LG11 | 0 | 0.5 | 0.09 | 0 | 0 | 0.32 |
| LG12 | 0 | 0.16 | 0.03 | 0.01 | 0 | 0.02 |
| **total proportion of distorted markers** | **0.04** | **0.26** | **0.08** | **0.04** | **0.02** | **0.05** |
| **total number of distorted markers** | **71** | **575** | **196** | **100** | **26** | **78** |
